# Clozapine-induced transcriptional changes in the zebrafish brain

**DOI:** 10.1038/s41537-019-0092-x

**Published:** 2020-02-03

**Authors:** Joana Viana, Nick Wildman, Eilis Hannon, Audrey Farbos, Paul O’ Neill, Karen Moore, Ronny van Aerle, Greg Paull, Eduarda Santos, Jonathan Mill

**Affiliations:** 1 University of Exeter Medical School, University of Exeter, Exeter, UK; 2 Biosciences, College of Life & Environmental Sciences, University of Exeter, Exeter, UK; 3 International Centre of Excellence for Aquatic Animal Health, Cefas Weymouth Laboratory, Weymouth, Dorset, UK; 4 Sustainable Aquaculture Futures, University of Exeter, Exeter, UK

**Keywords:** Schizophrenia, Molecular neuroscience

## Abstract

Clozapine is an atypical antipsychotic medication that is used to treat schizophrenia patients who are resistant to other antipsychotic drugs. The molecular mechanisms mediating the effects of clozapine are not well understood and its use is often associated with severe side-effects. In this study, we exposed groups of wild-type zebrafish to two doses of clozapine (‘low’ (20 µg/L) and ‘high’ (70 µg/L)) over a 72-h period, observing dose-dependent effects on behaviour. Using RNA sequencing (RNA-seq) we identified multiple genes differentially expressed in the zebrafish brain following exposure to clozapine. Network analysis identified co-expression modules characterised by striking changes in module connectivity in response to clozapine, and these were enriched for regulatory pathways relevant to the etiology of schizophrenia. Our study highlights the utility of zebrafish as a model for assessing the molecular consequences of antipsychotic medications and identifies genomic networks potentially involved in schizophrenia.

## Introduction

Schizophrenia is a severe psychiatric disorder affecting more than 21 million people worldwide and contributing significantly to the global burden of disease.^1,2^ It is characterised by symptoms of social isolation, apathy and lack of drive, interference with thought processes, hallucinations and delusions.^3^ There is no cure for schizophrenia; current treatments act by reversing the symptoms of disease rather than preventing the development of underlying pathology. These medications have relatively low efficacy for treating cognitive impairment and are not an efficient therapeutic of long term disability given the severe side-effects often associated with their use.^4^ Between 5% and 25% of schizophrenia patients are resistant to the most commonly prescribed antipsychotic medications,^5^ and clozapine is a second-generation antipsychotic often prescribed to patients with such treatment-resistant schizophrenia. Although clozapine has been shown to have increased effectiveness compared to other commonly prescribed antipsychotic drugs,^6,7^ over 40% of patients treated with clozapine show an inadequate response^8^ and its use is associated with severe side-effects. For example, patients prescribed clozapine need to be carefully monitored for the development of serious blood disorders such as agranulocytosis.^9^ The molecular mechanisms by which clozapine acts to alleviate the symptoms of schizophrenia are poorly understood.^10^ Identifying clozapine-related gene expression changes would help in understanding the mechanisms and side-effects of clozapine, and potentially identify molecular pathways and gene networks involved in schizophrenia.

To date, few studies have characterised gene expression changes in the brain associated with exposure to clozapine. Duncan and colleagues identified several differentially expressed genes in the mouse brain following clozapine exposure, including genes encoding potassium channel subunits.^11^ Another study identified clozapine-induced expression changes in the mouse striatum and cortex, including genes involved in neurotransmission, calcium homoeostasis and signal transduction.^12^ A study in rats identified clozapine-associated changes in the expression of genes involved in pathways, such as protein metabolism, nucleotide metabolism and signal transduction.^13^ Finally, Rizig and colleagues identified changes in the expression of glutamatergic, GABAergic, serotonergic, retinoid and G-coupled receptor genes in the mouse brain following clozapine exposure.^14^ Despite these preliminary findings, transcriptional responses to clozapine exposure have not been fully characterised.

Zebrafish (*Danio rerio*) is a widely used vertebrate model in neuroscience research^15^; there are multiple advantages of using zebrafish as a model, including high organizational and physiological similarity with mammalian brain structures,^15^ considerable (>70%) genomic orthologuey with human,^16^ rapid development and ease of genetic and other experimental manipulations.^15^ Compared to other models, the use of zebrafish is cost- and space-efficient, allowing the generation of larger sample sizes compared to analyses of rodents.^15^ Zebrafish have been used to model traits associated with several human psychiatric disorders including aggression, attention-deficit hyperactivity disorder (ADHD), post-traumatic stress disorder (PTSD), addiction and schizophrenia.^17,18^ Similarly, zebrafish models have been useful in tracking behavioural changes in response to epilepsy treatments,^19^ identifying activators of a metabolic fasting response^20^ and determining the effectiveness of commonly used medication in the treatment of Parkinsonian-like symptoms.^21^

In this study, we exposed zebrafish to two doses of clozapine and assessed behavioural and gene expression changes. We used RNA sequencing (RNA-seq) and co-expression network analysis to identify transcriptomic-wide changes in the zebrafish brain and modules of co-expressed genes sensitive to clozapine exposure. Our study highlights the utility of zebrafish as a model for assessing the molecular consequences of antipsychotic medications and identifies genomic networks potentially involved in schizophrenia.

## Results

### Overview of experimental approach

We exposed groups of wild type zebrafish to two doses of the antipsychotic drug clozapine (‘‘low’’ (20 µg/L) and ‘high’ (70 µg/L)) over a 72-h period. We assessed individual fish (including during spawning and feeding) to analyse changes in behaviour associated with clozapine exposure. Total RNA was isolated from brain tissue and used for messenger RNA-sequencing (RNA-seq) for samples exposed to water control (*n* = 16), dimethyl sulfoxide (DMSO) vehicle (*n* = 16), ‘low’’ clozapine exposure (*n* = 16) and ‘high’ clozapine exposure (*n* = 16). After stringent quality-control and pre-processing (see Methods), the RNA-seq data were used for differential gene expression analysis to identify clozapine-induced expression changes. Network analysis was performed to identify modules of co-expressed genes sensitive to clozapine exposure and gene ontology (GO) analysis was used to identify pathways and functions enriched in each of the clozapine exposure-associated modules. Supplementary Table 1 summarises phenotypic measures and RNA quality data for each of the individual samples used in this study. A schematic overview of the study is given in Supplementary Fig. 1 and additional details are provided in the Methods.

### Clozapine exposure had no general effects on fish health

We measured the body weight (g), fork length (cm), liver weight (mg) and brain weight (mg) and calculated the condition factor (k), hepatosomatic index (HSI) and brain-body ratio (BBR) for each individual fish (see Methods and Supplementary Table 1). None of these metrics differed significantly between groups (*P* > 0.05) (Supplementary Table 2), indicating that neither clozapine nor vehicle (DMSO) is associated with changes in any of these physiological characteristics under the experimental conditions tested in this study.

### Clozapine exposure induces striking behaviour effects in zebrafish

**Fig. 1:**
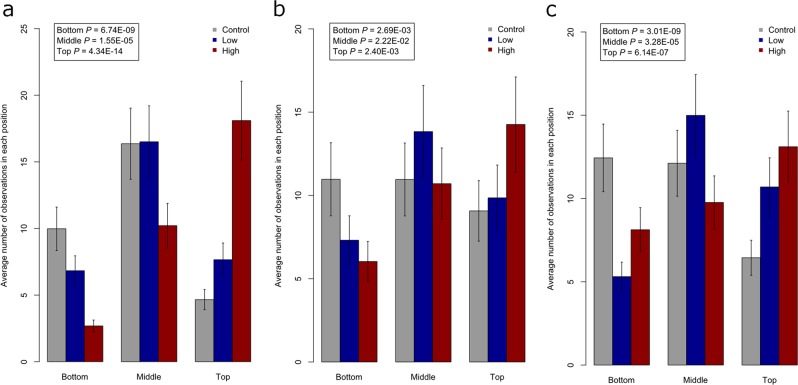
Clozapine exposure is associated with behaviour changes of zebrafish in the tank. Clozapine exposure induced a significant dose-dependent increase in the time spent at the top of the tank during **a** ‘general time’, **b** feeding and **c** spawning. All *P*-values < 2.78E-03 (Bonferroni corrected *P*-value for the 18 tests). The error bars represent the standard deviation.

We did not identify any significant behavioural differences between the water and DMSO groups at any of the time-points tested (Supplementary Table 3A). In contrast, we found that clozapine exposure had a significant dose-dependent effect on tank position during each of the behavioural phases (‘general time’ (Fig. 1a), feeding (Fig. 1b) and spawning (Fig. 1c), all *P* < 2.78E-03 (Bonferroni corrected *P*-value for the 18 tests)) (Supplementary Table 3B), with the ‘high’ exposure group spending a much larger proportion of time at the top of the tank. Conversely, clozapine exposure was associated with less time spent at the bottom of the tank during each of the three assessment periods (all *P* < 2.78E-03). Changes in the proportion of time spent in the middle of the tank were less pronounced, with clozapine exposure having a significant effect during ‘general time’ and spawning, but not feeding. Representative videos of clozapine-induced changes in tank position during ‘general time’ can be seen online at 10.6084/m9.figshare.9638900 (DMSO exposure) and 10.6084/m9.figshare.9638903 (‘high’, 70 µg/L exposure). The original videos are also available online (see Methods). Clozapine exposed fish did not show any signs of respiratory or other types of stress that might explain the increased time spent at the top of the tank.

### Clozapine exposure is associated with the altered gene expression

**Fig. 2:**
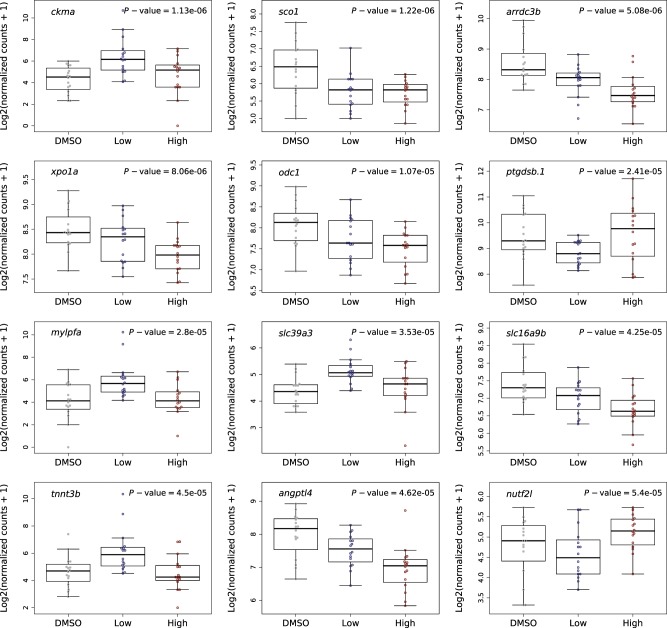
Clozapine exposure is associated with significant changes in the expression of twelve genes. Boxplots showing the expression levels of the twelve genes differentially expressed (FDR < 0.10) for the DMSO (grey), ‘low’ dose (20 µg/L clozapine; blue) and ‘high’ dose (70 µg/L clozapine; red) exposure samples. *P*-values are presented in each of the boxplots.

High-quality RNA was obtained from all individual fish included in this study (minimum, mean and maximum RNA integrity numbers (RIN) were 8.30, 9.37 and 10.00, respectively) (Supplementary Table 1). Following RNA sequencing and stringent quality control (QC) of the raw data (see Methods), we obtained an average of 33,089,096 (standard deviation (SD) = 8,636,715) quality-trimmed pair-ended reads per sample (Supplementary Table 4), with no difference in read-depth between exposure groups (*t*-test water vs DMSO *P* = 0.96 and ANOVA DMSO, 20 µg/L clozapine and 70 µg/L clozapine *P* = 0.25). In total, 20,837 genes were reliably detected and used for differential expression analysis (see Methods). To identify clozapine exposure-associated changes we performed a likelihood ratio test (LRT) using exposure (i.e. DMSO, 20 µg/L clozapine, and 70 µg/L clozapine), sex (male and female) and experimental week (first or second) as independent variables. Quantile-quantile (Q–Q) plots (Supplementary Fig. 2) and volcano plots (Supplementary Fig. 3A, B) of the association statistics from these analyses indicate that there are more striking differences between DMSO and clozapine than between water and DMSO. Overall results for each detectable gene are provided in the Supplementary Data file. Although two genes (*cremb* and *si:ch211-207b24.4*) showed expression differences between the water control and DMSO vehicle groups (FDR < 0.10) (Supplementary Data), neither of these was associated with exposure to clozapine. In total, the expression of twelve genes was significantly (FDR < 0.10) associated with clozapine exposure (Table 1 and Fig. 2). Both doses of clozapine exposure were associated with a reduction in the expression of five genes (*arrdc3b* (*P* = 5.05E-06), *xpo1a* (*P* = 8.12E-06), *odc1* (*P* = 1.07E-05), *slc16a9b* (*P* = 4.25E-05) and *angptl4* (*P* = 4.59E-05) (Fig. 2)), and an increase in expression of four genes (*ckma* (*P* = 1.11E-06), *mylpfa* (*P* = 2.77E-05), *slc39a3* (*P* = 3.54E-05) and *tnnt3b* (*P* = 4.46E-05) (Fig. 2)). The remaining differentially expressed genes were characterised by less consistent changes, with opposite-direction effects associated with ‘low’ and ‘high’ doses of clozapine (*xpo1a* (*P* = 8.12E-06), *ptgdsb.1* (*P* = 2.38E-05) and *nutf2l* (*P* = 5.36E-05)) (Fig. 2). All but two (*slc39a3* and *nutf2l*) of these differentially expressed genes have one (or more) known human orthologues (Table 1), many playing a role in functional pathways relevant to schizophrenia (see Discussion). Of note, there was no evidence for any significant interaction between the gene expression responses to exposure and sex (Supplementary Figs 3C and 4), indicating that exposure to clozapine does not have differential effects on gene expression in males and females.

**Table 1:** Differentially expressed genes significantly associated with exposure to clozapine. Statistics for all detectable genes are given in Supplementary Table 5.

Gene ID	Gene symbol	Base mean read-counts	log2 fold change (Control vs ‘Low’)	log2 fold change (Control vs ‘High’)	*P*-value	FDR	Human homolog(s)
ENSDARG00000035327	*ckma*	85.99	2.82	1.06	1.11E-06	0.01	*CKM*
ENSDARG00000067593	*sco1*	66.10	−0.76	−0.66	1.22E-06	0.01	*SCO1*
ENSDARG00000036028	*arrdc3b*	282.33	−0.60	−0.84	5.05E-06	0.04	*ARRDC3*
ENSDARG00000078041	*xpo1a*	303.69	−0.21	−0.37	8.12E-06	0.04	*XPO1*
ENSDARG00000007377	*odc1*	220.10	−0.36	−0.42	1.07E-05	0.04	*ODC1*
ENSDARG00000027088	*ptgdsb.1*	745.87	−0.79	0.36	2.38E-05	0.08	*PTGDS; LCN6; LCN15; LCN9; LCN1; LCN10; OBP2A; AL355987.1; LCNL1; LCN8; OBP2B; PAEP*
ENSDARG00000053254	*mylpfa*	63.39	2.29	0.52	2.77E-05	0.08	*MYLPF*
ENSDARG00000101434	*slc39a3*	26.23	0.75	0.32	3.54E-05	0.09	*−*
ENSDARG00000104687	*slc16a9b*	134.57	−0.39	−0.57	4.25E-05	0.09	*SLC16A9*
ENSDARG00000068457	*tnnt3b*	70.05	2.01	0.27	4.46E-05	0.09	*TNNT3*
ENSDARG00000035859	*angptl4*	193.64	−0.43	−0.84	4.59E-05	0.09	*ANGPTL4*
ENSDARG00000014499	*nutf2l*	29.00	−0.22	0.49	5.36E-05	0.09	−

*FDR* false discovery rate

### Co-expression gene modules show dosage sensitive variation in connectivity following clozapine exposure

**Fig. 3:**
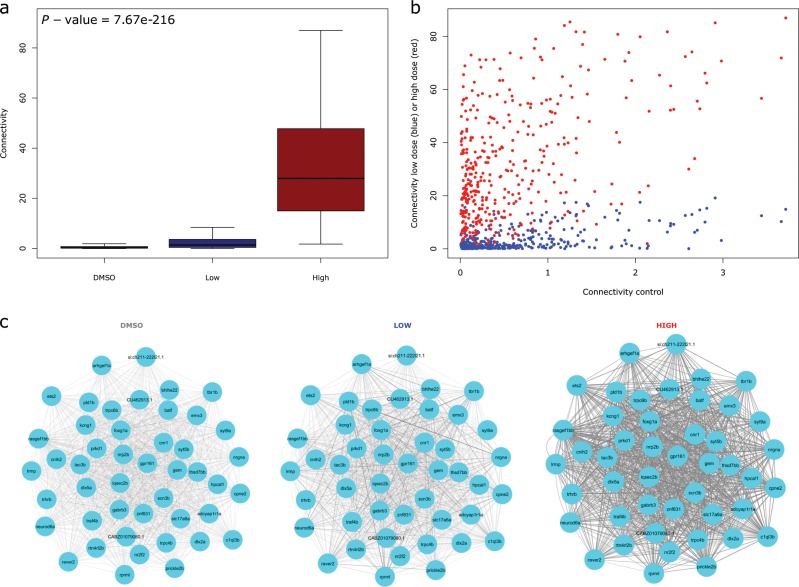
Example of a module characterised by increased connectivity between co-expressed genes following clozapine exposure. **a** There is a highly significant increase in connectivity within ‘Module 1’ following clozapine exposure, with the most dramatic increase seen following exposure to a ‘high’ dose of clozapine. **b** Connectivity for individual genes in ‘Module 1’ is notably higher following clozapine exposure relative to connectivity in the DMSO control group. **c** Visual representation of connectivity between individual genes in ‘Module 1’ following clozapine exposure (fifty genes with higher connectivity), highlighting the clear dose-response relationship. Thicker lines denote higher connectivity.

**Fig. 4:**
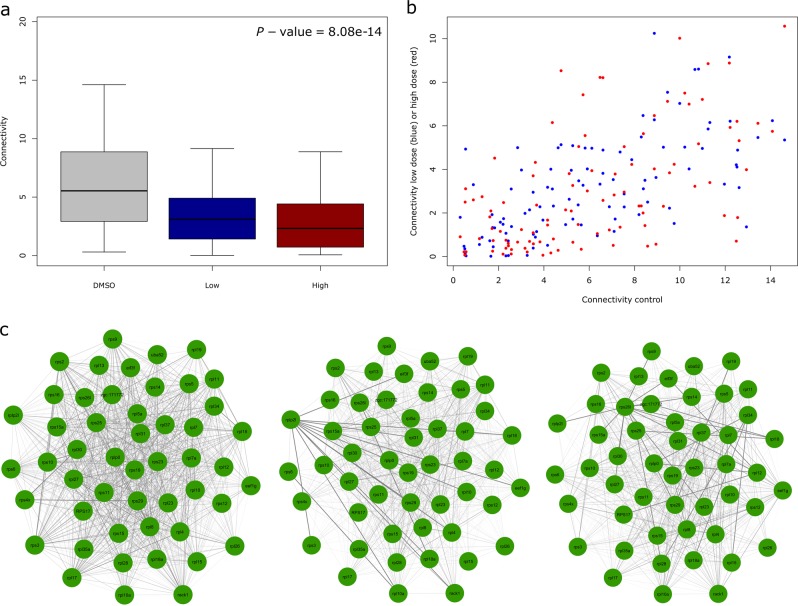
Enrichment of functional pathways related to G-protein-coupled receptor activity in ‘Module 1’, which is characterised by dramatically elevated gene connectivity following clozapine exposure. The applications ‘EnrichmentMap’ (v3.2.0) and AutoAnnotate’ (v1.3) in Cytoscape (v3.7.1) were used to generate this figure. GO terms are ordered by enrichment *P*-value with smaller numbers representing smaller *P*-values. GO terms were clustered by ‘gene set description’ (‘EnrichmentMap’). Overrepresented GO terms are circled with a red border (Bonferroni *P* < 3.34E-05, see Supplementary Table 6). The width of the edges is proportional to the number of genes in common between GO terms.

**Fig. 5:**
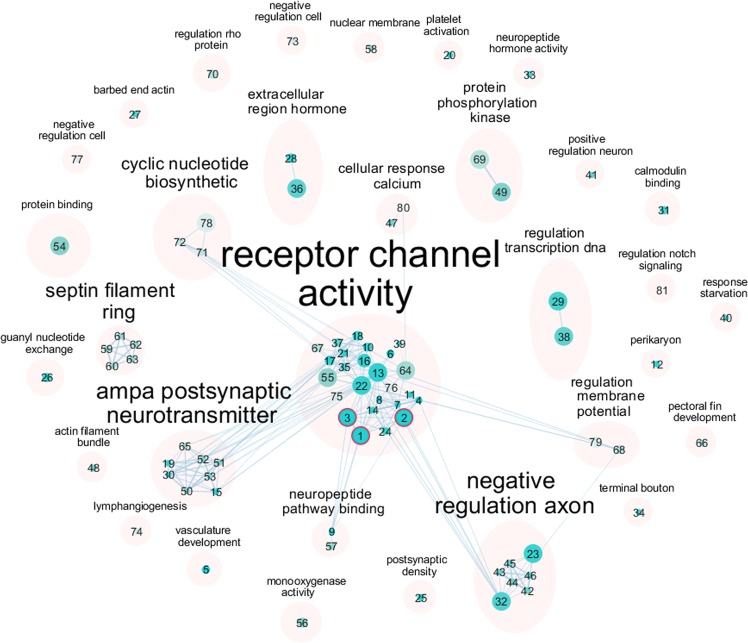
Example of a module characterised by decreased connectivity between co-expressed genes following clozapine exposure. **a** There is a highly significant decrease in connectivity within ‘Module 5’ following clozapine exposure, with the most dramatic decrease seen following exposure to a ‘high’ dose of clozapine. **b** Connectivity for individual genes in ‘Module 5’ is lower following clozapine exposure relative to connectivity in the DMSO control group. **c** Visual representation of connectivity between individual genes in ‘Module 5’ following clozapine exposure (50 genes with higher connectivity), highlighting the clear dose-response relationship.

We next used weighted gene co-expression network analysis (WGCNA) to explore system-level changes in zebrafish brain gene expression following clozapine exposure (see Methods). We created a network of co-expressed genes using RNA-seq data from all samples exposed to vehicle (DMSO) (*n* = 16), ‘low’ clozapine exposure (*n* = 16) and ‘high’ clozapine (*n* = 16), in total identifying 34 discrete co-expression modules (Table 2). Of these 20 were characterised by significant changes in intramodular connectivity (the sum of connection strengths with the other module genes, i.e. the extent to which genes within the same module are connected to each other) in response to clozapine exposure (Bonferroni corrected *P* = 1.47E-03, accounting for the total number of modules tested). Table 2 shows the average intramodular connectivity within each module for each exposure group, highlighting the extent to which different modules show varying patterns of altered connectivity after clozapine exposure. We investigated whether there was an over-representation of specific Gene Ontology (GO) categories in each of the clozapine-associated co-expression modules. Ten of the clozapine-associated modules had at least one overrepresented GO term (Bonferroni *P* < 3.34E-05, corrected for 1497 GO terms present in all the modules tested) (Supplementary Table 5), with many of the modules highly enriched for pathways directly related to schizophrenia and the pharmacology of clozapine. The largest module (‘Module 1’), which comprises 380 genes, was characterised by a dramatic increase in connectivity associated with exposure to clozapine (mean connectivity control = 0.59, mean connectivity ‘low’ clozapine exposure = 3.02, mean connectivity ‘high’ clozapine exposure = 32.43, ANOVA *P* = 7.67E-216, Fig. 3 and Table 2); in essence this reflects the coordinated upregulation of a discrete co-expressed set of genes in response to clozapine. ‘Module 1’ is enriched for genes involved in G-protein-coupled receptor (GPCR) activity (*P* *=* 1.56E-6) (Fig. 4 and Supplementary Table 5); this is interesting given the known interaction between clozapine and several G-protein-coupled receptors (GPCRs) including the dopamine D2 receptor.^22,23^ ‘Module 5’, in contrast, is characterised by a significant decrease in connectivity after clozapine exposure (mean connectivity control = 6.09, ‘low’ clozapine exposure = 3.18, ‘high’ clozapine exposure = 3.01, ANOVA *P* *=* 8.08E-14, Fig. 5 and Table 2); this reflects a module of co-expressed genes that is disrupted following clozapine exposure, with GO analysis showing it to be enriched for genes related to cytoplasmic translation (*P* < 2.93E-98, Supplementary Fig. 7 and Supplementary Table 5). The connectivity values for the remaining clozapine-associated modules are shown in Supplementary Fig. 5 with enriched GO categories shown in Supplementary Table 5 and Supplementary Figs 6–13.

**Table 2:** Co-expression gene modules associated with clozapine exposure.

		Connectivity				
Module	Number of genes	ANOVA *P*	Mean connectivity (control)	Mean connectivity (‘low’)	Mean connectivity (high)	GO terms over-represented
**1**	**380**	**7.67E-216**	**0.59**	**3.02**	**32.43**	**3**
**2**	**324**	**2.88E-155**	**2.36**	**1.12**	**22.02**	**0**
3	182	0.06	13.72	15.42	12.00	–
**4**	**105**	**5.41E-08**	**3.81**	**1.89**	**2.81**	**12**
**5**	**99**	**8.08E-14**	**6.09**	**3.18**	**3.01**	**17**
**6**	**79**	**4.25E-06**	**2.63**	**1.36**	**1.87**	**0**
**7**	**73**	**5.28E-32**	**0.70**	**1.09**	**4.16**	**2**
**8**	**69**	**8.38E-35**	**1.37**	**0.60**	**5.09**	**7**
**9**	**57**	**4.70E-52**	**0.60**	**0.03**	**7.76**	**0**
10	55	0.14	3.06	4.57	3.60	−
**11**	**49**	**1.35E-04**	**2.53**	**2.35**	**1.07**	**10**
**12**	**42**	**3.81E-07**	**0.60**	**1.42**	**1.06**	**0**
**13**	**41**	**1.01E-04**	**0.73**	**1.37**	**1.89**	**8**
14	41	0.11	1.72	1.33	2.04	−
15	37	0.03	1.40	1.32	0.84	−
**16**	**35**	**1.87E-14**	**0.19**	**1.35**	**2.26**	**9**
17	33	0.21	1.87	2.24	1.54	−
18	33	5.05E-04	2.64	3.47	1.52	−
**19**	**31**	**4.55E-10**	**1.40**	**0.14**	**2.45**	**4**
**20**	**31**	**2.77E-08**	**0.77**	**5.00**	**3.90**	**0**
21	30	1.22E-03	1.56	2.16	3.57	−
**22**	**27**	**9.34E-17**	**0.34**	**0.60**	**5.32**	**0**
23	26	0.99	3.00	2.87	2.98	−
24	24	0.11	1.65	2.59	3.09	−
**25**	**24**	**2.61E-04**	**2.72**	**3.24**	**1.13**	**3**
26	24	0.99	3.11	3.02	3.03	−
27	23	0.02	1.51	0.64	1.52	−
28	23	0.23	2.74	3.72	2.29	−
**29**	**23**	**2.94E-04**	**0.98**	**1.91**	**3.13**	**0**
**30**	**22**	**7.34E-05**	**1.47**	**0.50**	**2.99**	**0**
**31**	**22**	**5.67E-05**	**2.01**	**1.49**	**3.96**	**0**
32	22	0.04	1.56	2.76	1.48	−
**33**	**21**	**2.91E-19**	**0.10**	**0.15**	**4.21**	**0**
34	20	0.48	2.89	2.37	1.95	–

Modules are listed ranked by size. 20 modules (depicted in bold in the table) were characterised by a significant change in intramodular connectivity associated with clozapine exposure (Bonferroni corrected *P*-value = 1.47E-03, accounting for the total number of modules tested). Enriched gene ontology (GO) terms for each module are presented in Supplementary Table 5. Rows in bold depict significant modules associated with clozapine exposure

## Discussion

In this study we examined behavioural and transcriptional responses to clozapine exposure. First, we found that clozapine had a significant dose-dependent effect on swimming behaviour, with elevated exposure associated with a dramatic increase in the proportion of time spent at the top of the tank. Previous studies have shown that clozapine induces specific behavioural profiles in larval zebrafish.^24,25^ Furthermore, our findings corroborate those from a recent study that showed that clozapine acts to rescue aberrant swimming behaviour in zebrafish lacking the dopamine transporter gene which normally hover near the tank bottom.^26^ Second, our brain RNA-seq analyses identified altered expression of several genes in response to clozapine exposure. Several of the individual genes influenced by clozapine changed additively with clozapine dose, although for other genes the exposure-associated changes were more complex with opposite directions of differential expression in the low and high exposure groups; although the mechanism underlying these differences are not known, they are interesting given previous work highlighting differential effects of low- and high-doses in mice.^27^ Finally, we observed striking shifts in the connectivity between co-expressed genes in modules enriched for functions related to schizophrenia and the known pharmacology of clozapine. To our knowledge this represents the most systematic analysis of gene expression changes in the brain associated with exposure to clozapine, prioritizing genes and molecular pathways for future studies of schizophrenia and antipsychotic medications.

Several human orthologues of the individual zebrafish genes associated with clozapine exposure in this study are noteworthy for their hypothesised role in schizophrenia and/or the action of clozapine. For example, *CKM* (orthologue of *ckma*, which was upregulated following clozapine exposure) encodes a creatine kinase that catalyzes the phosphorylation of creatine in muscle. The expression of the heterodimer CKMB consisting of one muscle (CKM) and one brain-type (CKB) subunit has been shown to be a robust biomarker for acute myocardial infarction,^28^ an interesting observation given the elevated risk of myocarditis following clozapine exposure.^29^ Of note, elevated levels of serum CKM have been reported after both clozapine^30^ and olanzapine treatment.^31^ CKM is expressed in Purkinje neurons and is thought to be involved in unique calcium metabolism of these neurons and their role in cerebellar motor learning.^32,33^ Also of interest in the context of clozapine-induced side effects is *ANGPTL4* (an orthologue of *angptl4*, which was downregulated in response to clozapine); this gene is associated with triglyceride and high-density lipoprotein cholesterol levels^34^ and might be relevant to the elevated risk of type 2 diabetes in schizophrenia patients prescribed clozapine.^35^

Also noteworthy is *SCO1* (orthologue to *sco1*, which was downregulated following clozapine exposure). This gene encodes *cytochrome C oxidase assembly protein 1*, a copper metallochaperone that is essential for the maturation of cytochrome c oxidase subunit II and plays an important role in the regulation of copper homoeostasis.^36^ Copper plays a key role in functions such as mitochondrial activity and myelination^37^ and is transported from the bloodstream across the blood–brain barrier into astrocytes and then neurons via the copper uptake protein 1 (CTR1).^38^ Inside the cell, copper is delivered to the trans-Golgi network and from there distributed to metallochaperones including SCO1, which transport it to the mitochondria.^39^ Schizophrenia patients have been shown to exhibit altered plasma copper levels^40^ and agents which decrease copper levels produce schizophrenia-like behavioural in rats.^41^ One possible hypothesis would be that clozapine regulates the abnormal levels of copper observed in schizophrenia by altering the expression of *SCO1*, however more work is needed to understand the mechanism of action.

Another interesting human orthologue is *ODC1* that encodes *ornithine decarboxylase 1* (orthologue to *odc1*, which is downregulated following clozapine exposure), an enzyme that catalyzes the conversion of ornithine into putrescine.^42^ Putrescine is a polyamine, together with spermine, and spermidine, and plays a major role in the regulation of cell growth and differentiation, metabolic pathways, and cell membrane functions in mammalian systems.^43^ It has been suggested that polyamines may be involved in the pathophysiology of schizophrenia and other mental health conditions.^44^ Ornithine, ODC1 and putrescine are involved in L-arginine metabolism,^45^ which has also been implicated in the pathogenesis of schizophrenia.^46–49^ Although studies in schizophrenia have found no differences in ODC1 levels or activity in three different brain regions,^50,51^ levels of plasma ornithine have been shown to be elevated in patients.^52^

Finally, our results also highlight *ARRDC3*, which encodes *Arrestin Domain Containing 3* (orthologue to *arrdc3b*, which is downregulated following clozapine exposure), an adapter protein that plays a role in regulating cell-surface expression of adrenergic receptors and other GPCRs.^53–56^ *ARRDC3* has also been shown to be differentially expressed in the prefrontal cortex in schizophrenia.^57^ This is an interesting finding since GPCRs play a major role in schizophrenia and its treatment.^22,23^ Further evidence for disrupted GPRC activity comes from our systems-level analysis, with the largest co-expression module (‘Module 1’) being highly enriched for genes related to GPRC activity (Supplementary Table 5) characterised by a dramatic increase in connectivity following clozapine exposure in a dose-dependent manner. Altered levels of several G-protein-coupled receptor kinases (GRKs), which are involved in GPCRs desensitisation, have been found previously after clozapine and haloperidol treatment.^58^ GPCRs are phosphorylated by GRKs and subsequent bind to arrestin, which stops receptor-G-protein interaction and induces receptor internalisation.^56^

Several other functional pathways are enriched amongst modules highlighted by our co-expression network analyses (Supplementary Table 6). ‘Module 4’ and ‘Module 13’, for example, are highly enriched for GO categories related to mitochondrial function, which is interesting given the evidence for a link between mitochondrial dysfunction and pathogenesis of schizophrenia,^59,60^ and data showing an effect of clozapine on mitochrondrial activity.^61,62^ This is also interesting considering the downregulation of *sco1* following clozapine exposure discussed above. Our data lend support to the hypothesis that impairments to mitochondrial activity underlie some of the metabolic side-effects (e.g. insulin resistance, hepatic steatosis and accelerated weight gain) of clozapine.^61,63^ Other functional pathways highlighted by our co-expression network analyses include several implicated in schizophrenia including axon development (‘Module 7’)^64^ and circadian processes (‘Module 11’).^65^ Overall, more dramatic changes in response to clozapine were identified at the co-expression network level highlighting the power of this approach for identifying changes in discrete gene pathways.

Although comparisons with previous studies of clozapine-induced gene expression changes in rodents are difficult given the different models, technologies and experimental strategies employed, it is interesting that several common pathways appear to be represented—for example calcium metabolism, signal transduction and lipid metabolism which were also highlighted in a study on rat brain.^12^ There are, however, several limitations to this study that should be considered when interpreting our findings. First, despite the advantages of using zebrafish as a model for genomics and neuroscience,^15^ there are important differences between transcriptional control in zebrafish and human. Zebrafish are non-mammalian, with a less complex central nervous system (CNS) than humans, although they show similar patterns of brain development and conserved brain structure with mammals.^15,66^ Furthermore, there is a high degree of genetic orthologuey to human, with >70% of the genome conserved.^16^ Second, our analyses were performed on bulk brain tissue comprising a mix of brain regions and different neural cell-types. Future work should focus on transcriptionally profiling discrete populations of cells from specifically dissected brain regions. Third, we only assessed two acute exposures to clozapine; although this enabled us to dissect the primary response pathways following clozapine exposure without adaptive responses acting as a confounder, our analyses are not representative of the chronic polypharmaceutical exposures experienced by patients with schizophrenia. Finally, although our study represents the most systematic analysis of gene expression changes associated with clozapine yet undertaken, we only quantified gene expression in a relatively small number of individuals from an individual zebrafish strain and our findings warrant further replication.

In summary, we provide evidence for behavioural and gene expression changes in the brain—at both the single gene and systems level—following exposure to clozapine. Our study highlights the utility of zebrafish as a model for assessing the molecular consequences of antipsychotic medications and identifies genomic networks potentially involved in mediating the response to clozapine and the etiology of schizophrenia.

## Methods

### Fish husbandry

All animal procedures were conducted according to guidelines of the United Kingdom’s Home Office and approved by The University of Exeter Animal Ethics Committee. Wild type WIK strain adult zebrafish were maintained according to the conditions reported in Paull et al.^67^ (originating from a stock population (October 2014)). The experimental tanks used were 300 mm × 300 mm × 600 mm and were filled with 15 L of water. A flat constructed plastic base was placed in the bottom of each tank and opaque dividers were placed in between adjacent tanks to prevent visual interactions between fish in neighbouring tanks. Each tank was divided into three main areas using a grid (bottom, middle and top). Mains tap water was filtered by reverse osmosis (Environmental Water Systems (UK) Ltd.) and reconstituted with Analar-grade mineral salts to standardized synthetic freshwater (final concentrations to give a conductivity of 300 mS: 122 mg/L CaCl22H2O, 9.4 mg/L NaHCO3, 50 mg/L MgSO47H2O, 2.5 mg/L KCl, 50 mg/L Tropic Marin Sea Salt), aerated and heated to 28 °C in a reservoir, before it was supplied to each aquarium using a flow-through system. Tanks were aerated and supplied with a flow rate of 48 L/day of water maintained at 28 ± 0.5 °C and pH 7–7.5. Fish were maintained under a 12 h light:dark cycle, including dawn and dusk transition periods of 30 min and were fed live *Artemia nauplii* (ZM Premium Grade Artemia; ZM Ltd.) once daily day at 11.00 a.m., except on the final day prior to sampling. Fish were maintained in large mixed-sex holding tanks prior to the experiment and two males and two females were randomly allocated into each experimental tank on day 1 of the experiment (Supplementary Fig. 1 shows a diagram of the experiment).

### Exposure of zebrafish to clozapine

Fish were exposed to two different concentrations of clozapine (‘low’ concentration: 20 µg/L and ‘high’ concentration: 70 µg/L) and dimethyl sulfoxide (DMSO; 70 µl/L) was used as a vehicle to dissolve clozapine. DMSO and water control were also used in parallel.: 24 h after the introduction of the fish to the exposure tanks, to allow for acclimation, the chemical exposure was initiated by adding pre-prepared solutions of clozapine to each tank to reach the desired concentration (two tanks per condition), and initiation of a flow through exposure system in which appropriate concentrations of stock solutions of clozapine, DMSO or water were pumped into each tank using a peristaltic pump to maintain the exposure concentrations and water flow during the exposure period. Flow rates were monitored daily to ensure the chemical concentrations remained consistent. Fish were fed twice daily, once with live Artemia *nauplii* (ZM Premium Grade Artemia; ZM Ltd.) and another time with TetraMin tropical flake food (Tetra; Melle, Germany), to satiation. Stock solutions were replaced daily. After 72 h of exposure, all fish were sacrificed humanely using a lethal dose of benzocaine in accordance with UK Home Office regulations. The brains were dissected and weighed. All samples were snap frozen in liquid nitrogen and stored at −80 °C. The experiment was repeated in two consecutive weeks resulting in four independent tanks containing two males and two females per treatment. The total number of fish was 64, 16 per exposure group.

### Measures of fish health

During sampling, the wet weight and fork length were recorded, and the condition factor was calculated (k = [body weight (g) × 100]/[fork length (cm)]3). The hepatosomatic index (HSI = liver weight (mg)/[total weight (mg) − liver weight (mg)] × 100) and brain-body ratio (BBR = brain weight (mg)/[total weight (mg) − liver weight (mg)] × 100) were also calculated. These measurements can be found in Supplementary Table 1. To identify differences between the two control groups in each of the metrics (body weight, body length, k, HSI and BBR) we performed a linear regression in females and males separately using exposure (water and DMSO) and week of the experiment as independent variables. To identify differences in the same metrics between the experimental groups we performed one-way analysis of variance (ANOVA) in females and males separately, using exposure (DMSO, 20 and 70 µg/L) and week as independent variables. Statistical analyses were performed in R.^68^

### Behaviour measures

Each tank was videoed using an AXIS M1054 network camera (Axis Communications, Luton, Bedfordshire, UK) with a video resolution of 1280 × 800 pixels, coupled to a Synology network-attached storage device (NAS) (Synology Inc., Taipei, Taiwan). A laptop computer was used to connect to the NAS, via the network, to view the tank in real time and to record the tests and analyse behaviour. Each camera was clamped in front of each tank and these were recorded for from 9.15 a.m. to 9.45 a.m. (spawning), 11.00 a.m. to 11.30 a.m. (feeding) and 1.30 p.m. to 2.00 p.m. (‘general time’) during the three days of the exposure. For analysis, we paused the videos every minute, recorded the position of each fish (bottom, middle and top) and calculated the sum of the number of times each fish spent in each position over the 30 min. Analyses were performed separately for each position in the tank (bottom, middle and top) at each recording time (feeding, spawning and general time) and the three days of each experiment were treated as independent observations. To identify differences between the two control groups we performed a linear regression using exposure (water and DMSO), sex and time (week) of the experiment as independent variables. To identify clozapine exposure-associated differences we performed one-way ANOVA using exposure (DMSO, 20 and 70 µg/L), sex and time (week) of the experiment as independent variables. Statistical analyses were performed in R.^68^

### RNA sequencing

Total RNA was isolated from the whole brain samples using the AllPrep DNA/RNA Micro Kit (Qiagen, Venlo, Holland) incorporating on-column DNase treatment, according to the manufacturer’s instructions. RNA samples were assessed for quality and purity using spectrophotometry and an Agilent 2100 Bioanalyzer Instrument (Agilent Technologies, Santa Clara, CA, USA) in conjunction with the Agilent RNA 6000 Nano Kit (Agilent Technologies, Santa Clara, CA, USA) according to the manufacturer’s instructions. The RNA integrity numbers (RIN) ranged from 8.30 to 10.00 (Supplementary Table 1).

ERCC spike-in control mixes (Ambion, Thermo Fisher Scientific, Waltham, MA, USA) were added to all individual RNA samples, according to the manufacturer’s instructions. cDNA libraries from all samples were then prepared using the TruSeq Stranded mRNA Library Prep kit (Illumina, San Diego, CA, USA), multiplexed with eight samples per lane and RNA-sequencing was carried out on an Illumina HiSeq 2500 Sequencing System (Illumina, San Diego, CA, USA) to generate 100 bp paired reads.

### RNA-seq data pre-processing

Data pre-processing was performed in UNIX. The expression profiles of the ERCC sequences were analysed against the manufacturer’s expression values to check for accuracy of the transcript quantification and dynamic range. *Trimmomatic* (v0.38)^69^ was used to quality trim the raw reads. The raw and trimmed read were inspected using *FastQC* (v0.10.1).^70^ All samples survived quality control (QC) and pre-processing and were used for further analyses. *RSEM*^71^ (v1.3.1, using *bowtie2* v 2.3.4.1^72^) was used to align the pre-processed reads to the zebrafish complementary reference transcriptome (GRCz11 GCA_000002035.4) release 92, downloaded from Ensembl: ftp://ftp.ensembl.org/pub/release-92/fasta/danio_rerio/cdna/). The number of raw and trimmed reads, as well as the mapping efficiency for all samples is shown in Supplementary Table 4. The ‘abundance_estimates_to_matrix.pl’ script in *RSEM*^71^ was used to generate a matrix of estimated counts for all samples (30,627 genes).

### Differential expression analyses

Differential expression analyses were performed in R^68^ using the DMSO and clozapine (20 and 70 µl/L) samples. Genes were considered as being expressed when at least 10 reads in at least 16 samples (the exposure group size) were obtained and 20,837 genes were taken further for analysis. The ‘DESeq’ function from the R package *DESeq2* (v1.14.1)^73^ was used to perform the differential expression analyses. In total, 72 genes were identified as having outliers based on their Cook’s distance. The ‘DESeq’ function replaces these values with the trimmed mean over all samples (for details see Love et al.^73^). To identify clozapine exposure-associated gene expression changes we performed a likelihood ration test using exposure (DMSO, 20 and 70 µg/L), sex and week as independent variables. To see if clozapine dose interacts with sex, we repeated the analysis adding the interaction term ‘exposure*sex’. To identify changes between the two control groups (water and DMSO), the expression values of these two groups for the same 20,837 genes were extracted. We then performed a Wald test (‘DESeq’ function, *DESeq2*^73^) using exposure (water and DMSO), sex and week as independent variables.

### Co-expression network analysis

Network analysis on the gene expression values of the 20,837 genes that survived QC was performed using the R package *WGCNA* (v1.66).^74^ We created a signed network using the ‘blockwiseModules’ function (Pearson correlation, soft thresholding power = 12, minimum module size = 20, maximum block size = 5,000). The base 2 logarithms (log2) of the normalised counts + 1 of the DMSO, 20 µg/L and 70 µg/L groups were used. ‘Module 0’ containing non-allocated genes was excluded from further analysis. Connectivity values for each gene were calculated separately for the DMSO, 20 and 70 µg/L groups using the ‘intramodularConnectivity’ function on the absolute correlations between genes raised to the power of 12. We performed a one-way ANOVA using the ‘within module connectivity’ (kWithin) of the three exposure groups of interest (DMSO, 20 and 70 µg/L) to identify significant changes in module connectivity with clozapine exposure.

### Gene ontology pathway analysis

We tested for enrichment of GO^75,76^ terms in each of the modules showing connectivity changes with clozapine. The R annotation package *org.Dr.eg.db* (v 3.7.0)^77^ was used to extract updated GO terms for GRCz11 and terms with less than 10 genes were excluded from analyses. In total, 2,141 GO terms were assigned to genes in the dataset. The ‘makeTxDbFromBiomart’ function from the R package *GenomicFeatures* (v1.34.1)^78^ was used to extract the length of the genes in the analyses. For the genes allocated to each module, the ‘nullp’ and ‘goseq’ functions from the R package *goseq* (v1.34.0)^79^ were used to calculate the probability weighting function and test for GO term enrichment, respectively. For the results of each module, we displayed the results graphically using *Cytoscape* (v3.7.1).^80^

### Reporting summary

Further information on research design is available in the Nature Research Reporting Summary linked to this article.

## Supplementary information

Supplementary Information

Supplementary Data

Reporting Summary

## Data Availability

Raw, trimmed and pre-processed RNA-seq data as well as the original behavioural videos are available from https://osf.io/a97j8/ (10.17605/OSF.IO/A97J8). Differential gene expression results for all expressed transcripts is provided in the Supplementary Data file.

## Code availability

The code used to produce the analyses in this manuscript, as well as the phenotype file, can be found in https://git.exeter.ac.uk/jffpviana/ClozapineZebrafish.git.
