# Supplementary material for: Clozapine-induced transcriptional changes in the zebrafish brain
Source: NPJ Schizophr. 2020 Feb 3;6:3. doi: 10.1038/s41537-019-0092-x (PMC6997376; doi:10.1038/s41537-019-0092-x)
Supplement: Supplementary file 1 — Supplementary Information [file 41537_2019_92_MOESM1_ESM.pdf]

**Supplementary Information File**

**Supplementary Data Set 1 - Results of differential gene expression analysis following clozapine exposure for all detectable transcripts in zebrafish brain.** FDR – False Discovery Rate.

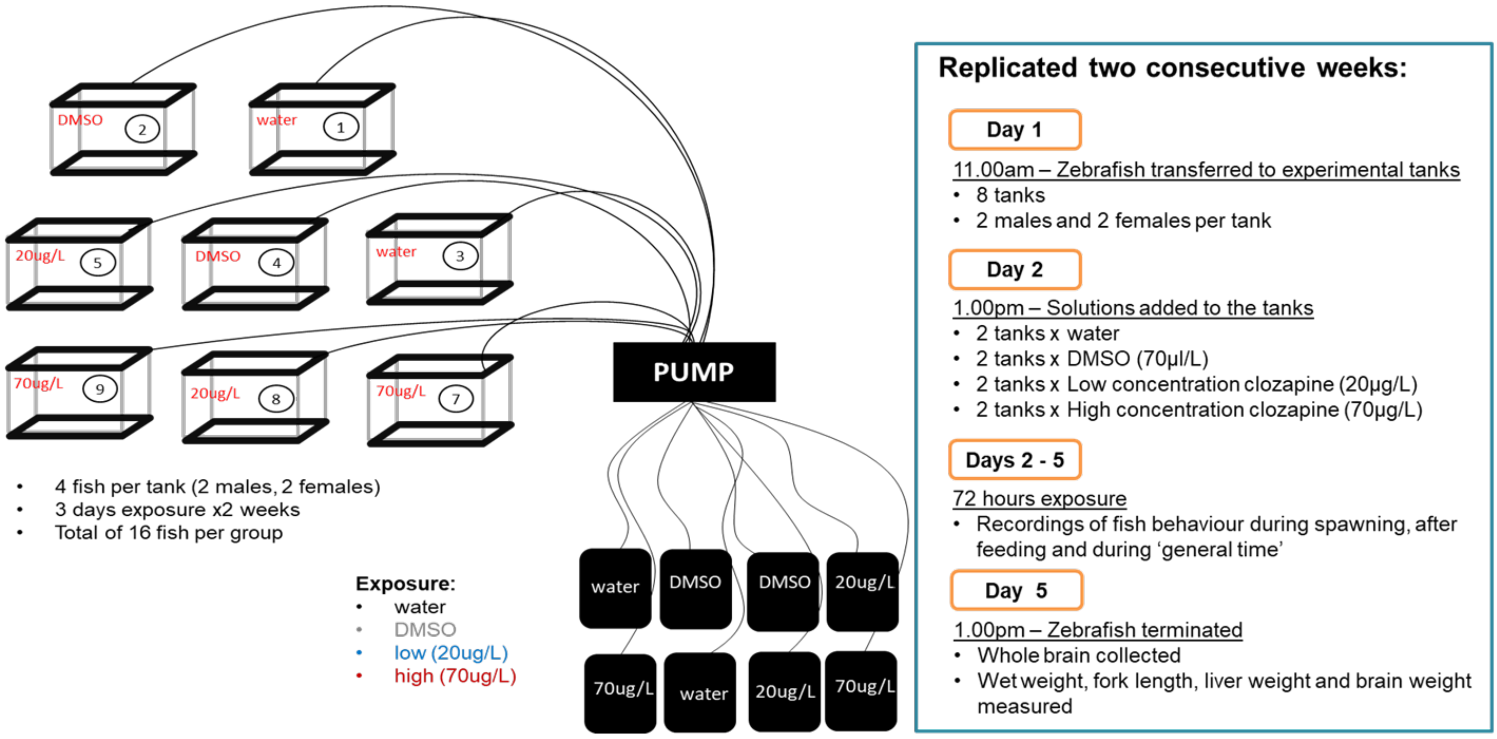


**Supplementary Figure 1 – An overview of the experimental design used in this study.**

A

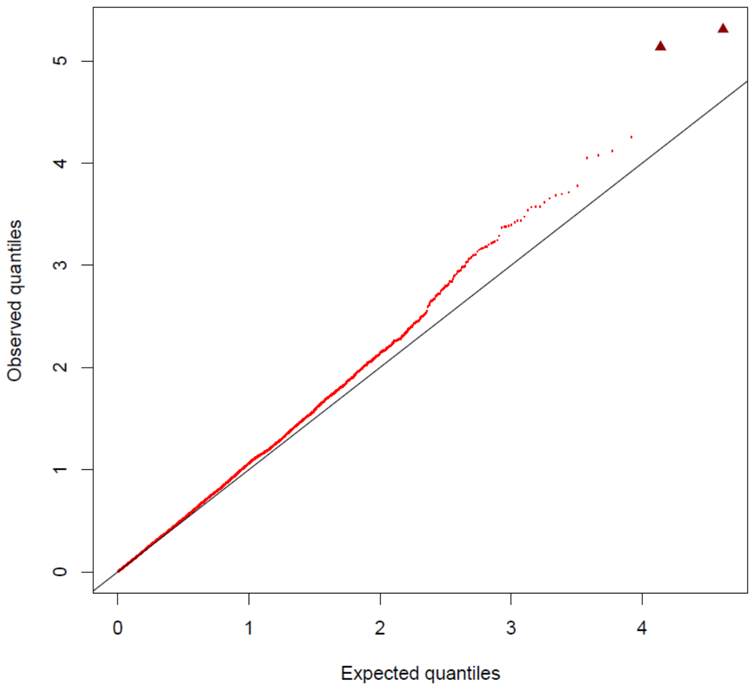


B

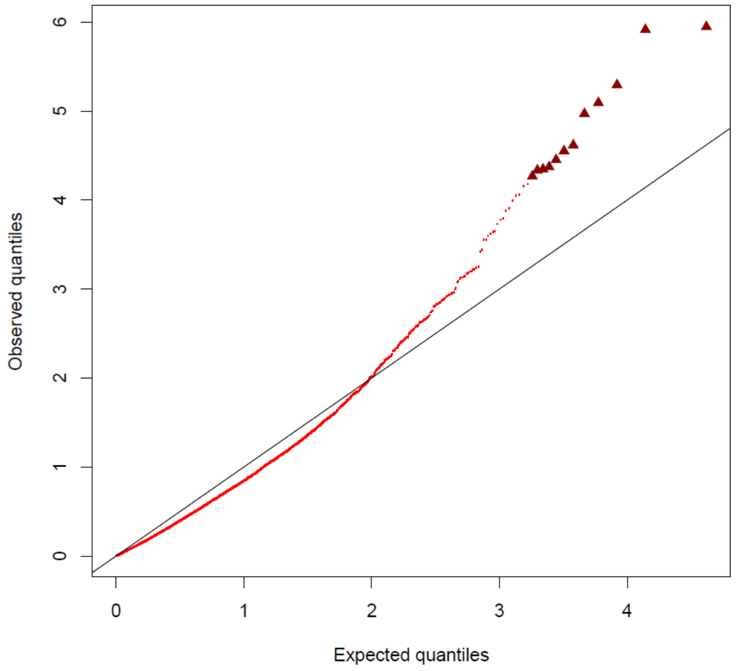


**Supplementary Figure 2 – Quantile-quantile (QQ) plot of association statistics from a model designed to identify gene expression differences between zebrafish exposed to A) water and DMSO and B) DMSO and clozapine**. Twelve transcripts were characterized by significant differences (FDR < 0.10) in gene expression associated with clozapine exposure. Results for all expressed genes are given in **Supplementary Table 4**. Triangles highlight transcripts with FDR < 0.10.

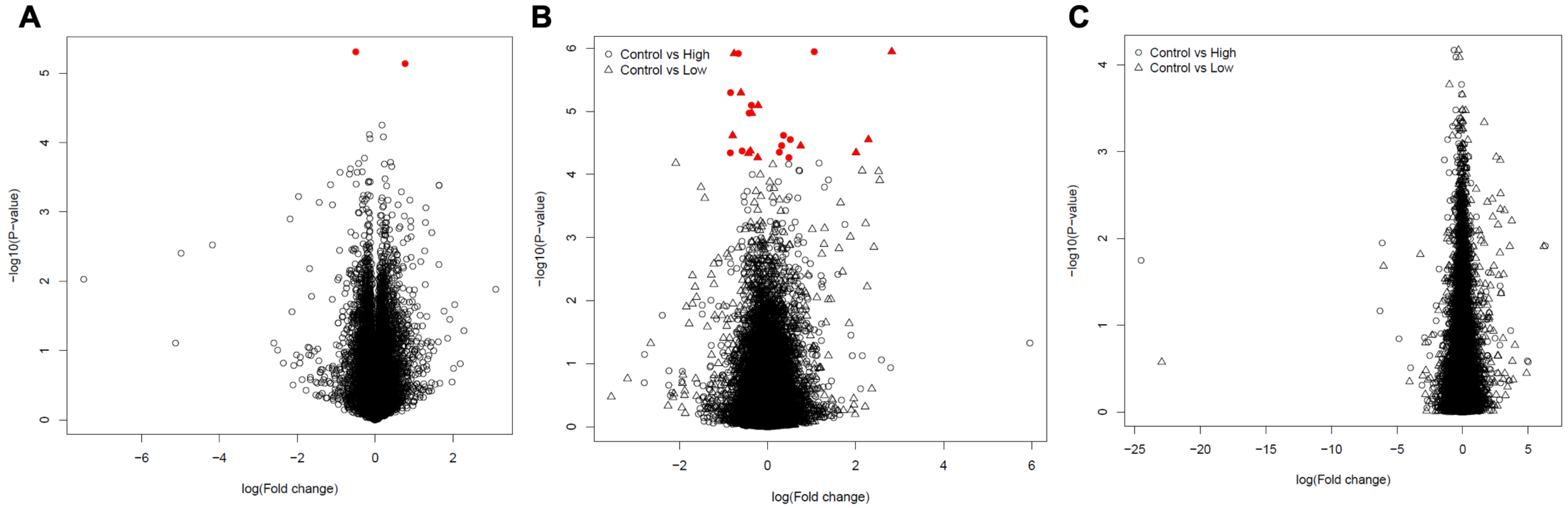


**Supplementary Figure 3 – Volcano plots highlighting association statistics for gene expression analyses of A) water vs DMSO, B) clozapine vs DMSO, and C) a test of interaction between sex and clozapine exposure.** Results for all expressed genes are given in **Supplementary Table 4**.

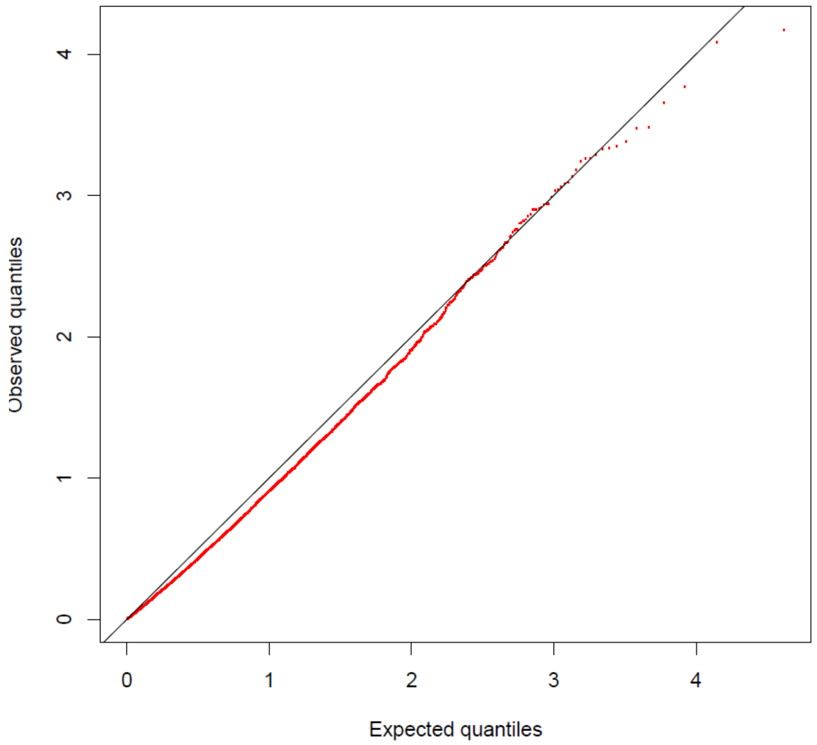


**Supplementary Figure 4 – Quantile-quantile (Q-Q) plot of association statistics from a model testing gene expression differences reflecting an interaction between clozapine exposure and sex.** No significant differences were identified, indicating that exposure to clozapine does not have differential effects on gene expression in males and females. Results for all expressed genes are given in **Supplementary Table 4**.

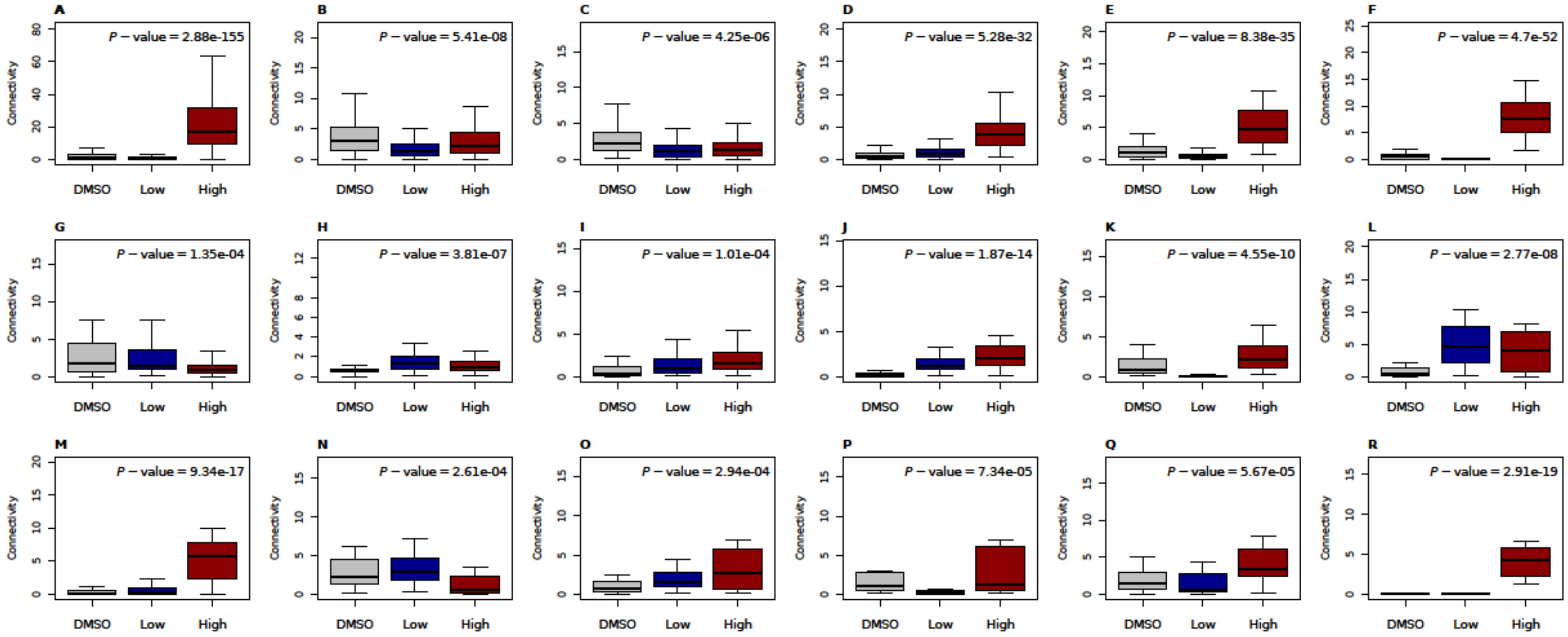


**Supplementary Figure 5 – Clozapine exposure is associated with altered transcript connectivity in multiple brain co-expression modules.** Shown is the relationship between mean connectivity and clozapine exposure for transcripts in eighteen additional modules. The results for two other modules ('Module 1' and 'Module 5') are shown in **Figure 3** and **Figure 4**, respectively.

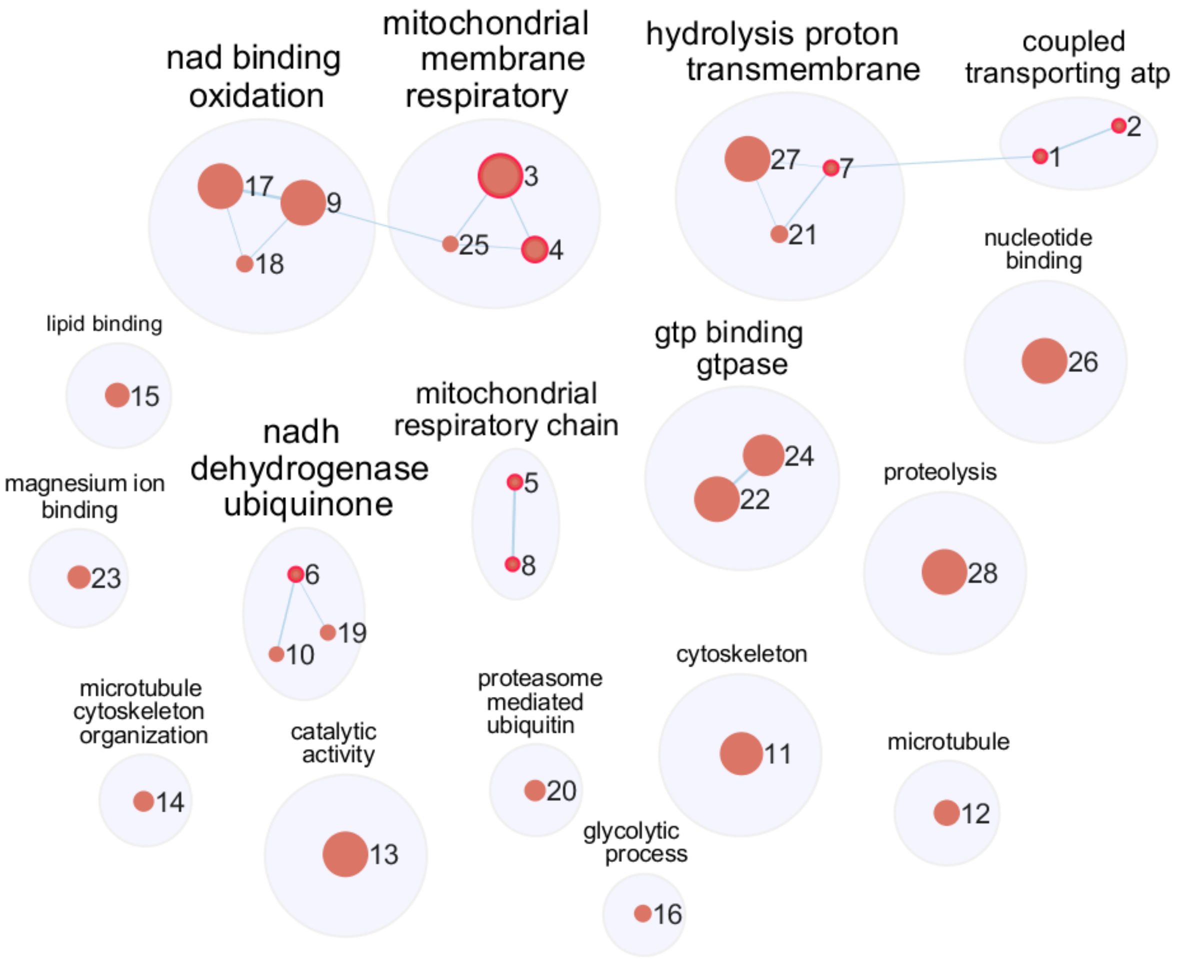


**Supplementary Figure 6 – Clusters of gene ontology (GO) categories enriched in 'Module 4'.** The applications 'EnrichmentMap'' (v3.2.0) and AutoAnnotate' (v1.3) in Cytoscape (v3.7.1) were used to generate this figure. GO terms are ordered by enrichment *P*-value with smaller numbers representing smaller *P*-values. GO terms were clustered by 'gene set description' ('EnrichmentMap'). Overrepresented GO terms are circled with a red border (Bonferroni *P* < 3.34E-05, see **Supplementary Table 6**). The width of the edges is proportional to the number of genes in common between GO terms.

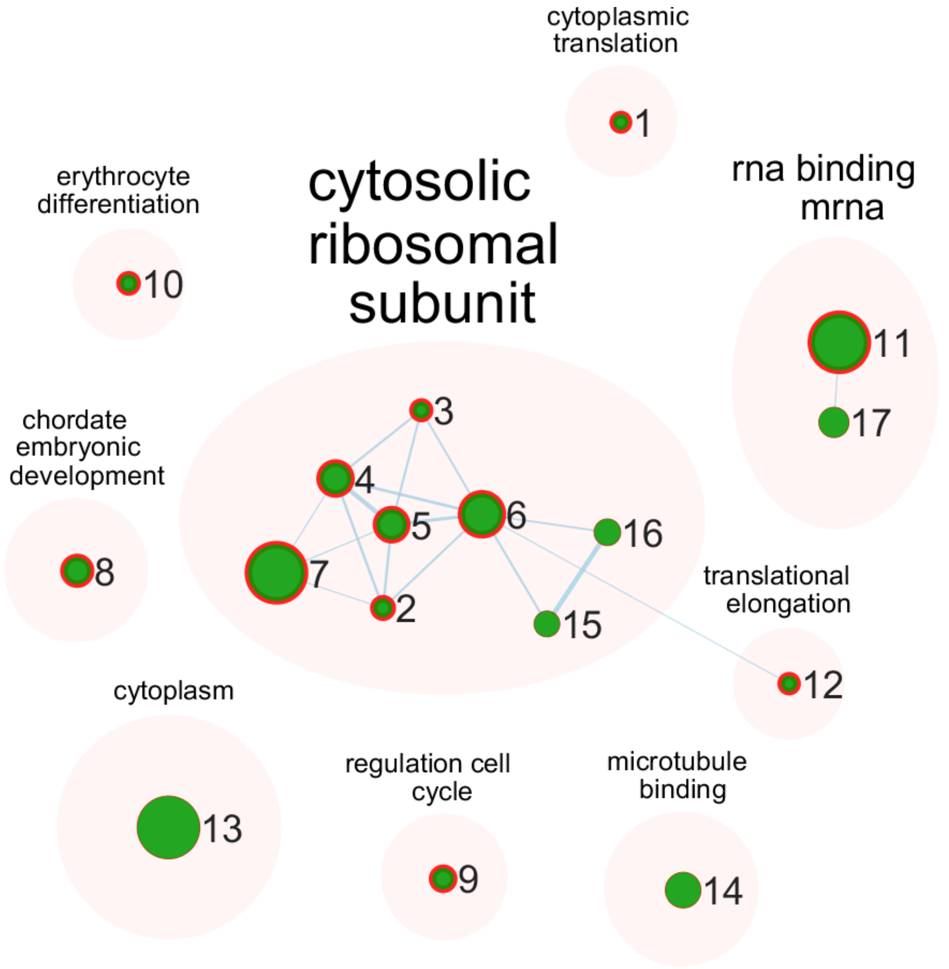


**Supplementary Figure 7 – Clusters of gene ontology (GO) categories enriched in 'Module 5'.** The applications 'EnrichmentMap'' (v3.2.0) and AutoAnnotate' (v1.3) in Cytoscape (v3.7.1) were used to generate this figure. GO terms are ordered by enrichment *P*-value with smaller numbers representing smaller *P*-values. GO terms were clustered by 'gene set description' ('EnrichmentMap'). Overrepresented GO terms are circled with a red border (Bonferroni *P* < 3.34E-05, see **Supplementary Table 6**). The width of the edges is proportional to the number of genes in common between GO terms.

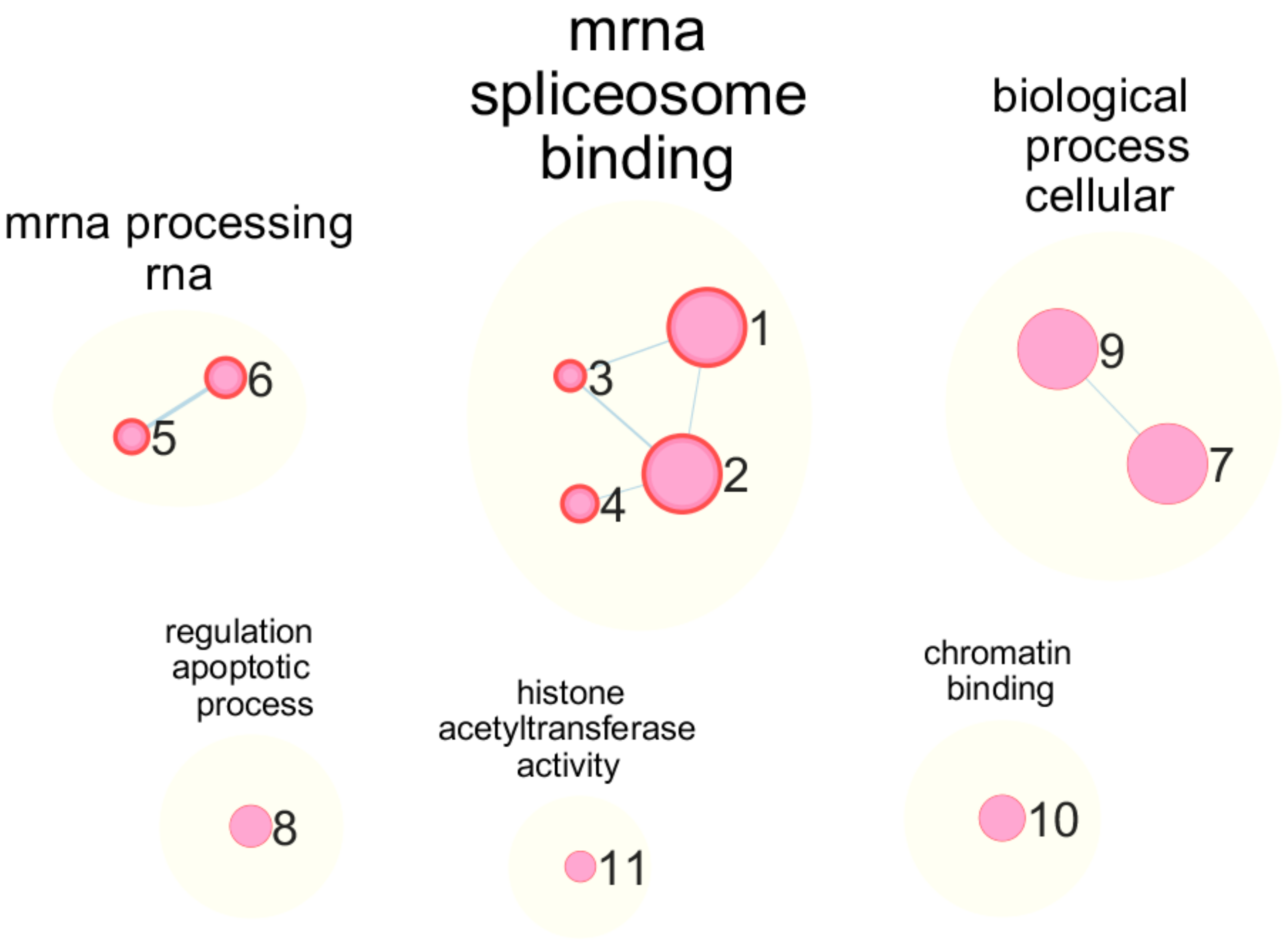


**Supplementary Figure 8 – Clusters of gene ontology (GO) categories enriched in ‘Module 8’.** The applications ‘EnrichmentMap’’ (v3.2.0) and AutoAnnotate’ (v1.3) in Cytoscape (v3.7.1) were used to generate this figure. GO terms are ordered by enrichment *P*-value with smaller numbers representing smaller *P*-values. GO terms were clustered by ‘gene set description’ (‘EnrichmentMap’). Overrepresented GO terms are circled with a red border (Bonferroni *P* < 3.34E-05, see **Supplementary Table 6**). The width of the edges is proportional to the number of genes in common between GO terms.

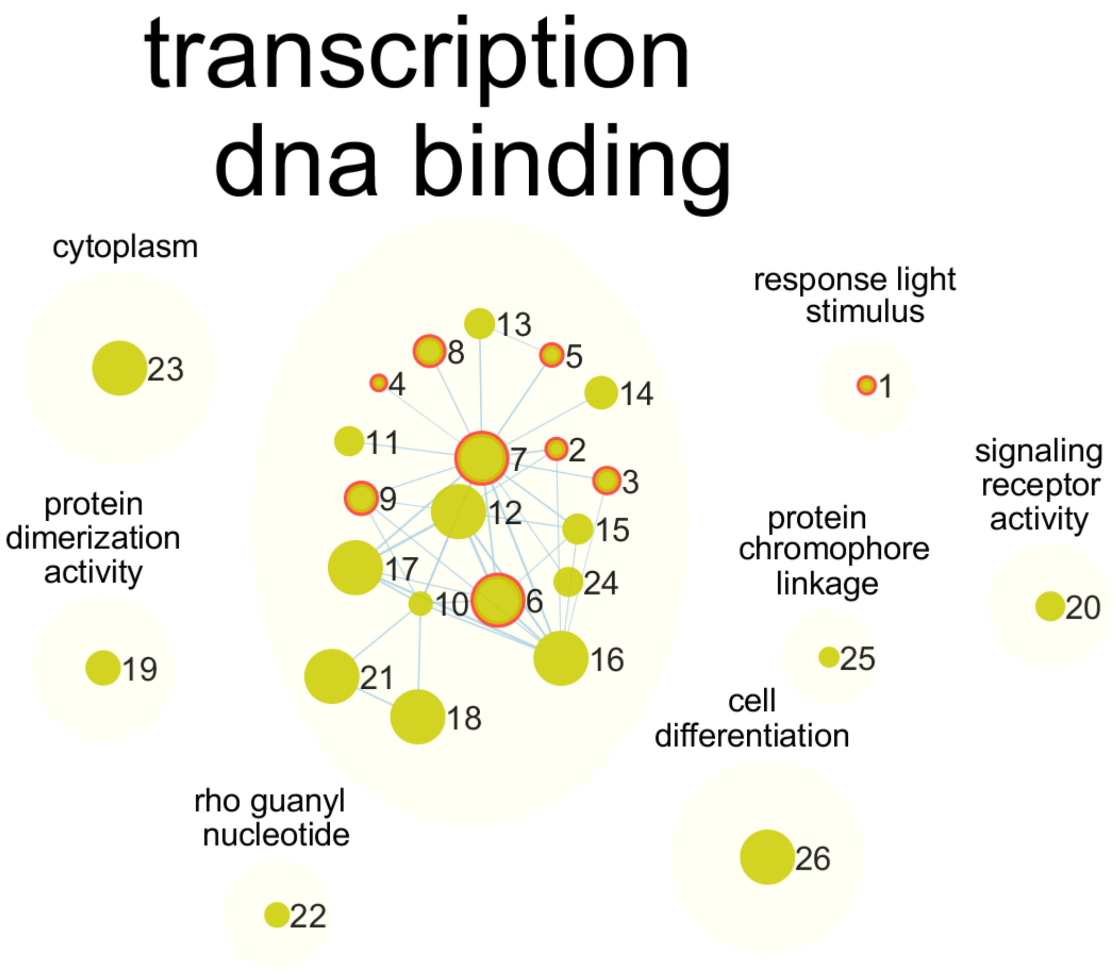


**Supplementary Figure 9 – Clusters of gene ontology (GO) categories enriched in 'Module 11'.** The applications 'EnrichmentMap'' (v3.2.0) and AutoAnnotate' (v1.3) in Cytoscape (v3.7.1) were used to generate this figure. GO terms are ordered by enrichment *P*-value with smaller numbers representing smaller *P*-values. GO terms were clustered by 'gene set description' ('EnrichmentMap'). Overrepresented GO terms are circled with a red border (Bonferroni *P* < 3.34E-05, see **Supplementary Table 6**). The width of the edges is proportional to the number of genes in common between GO terms.

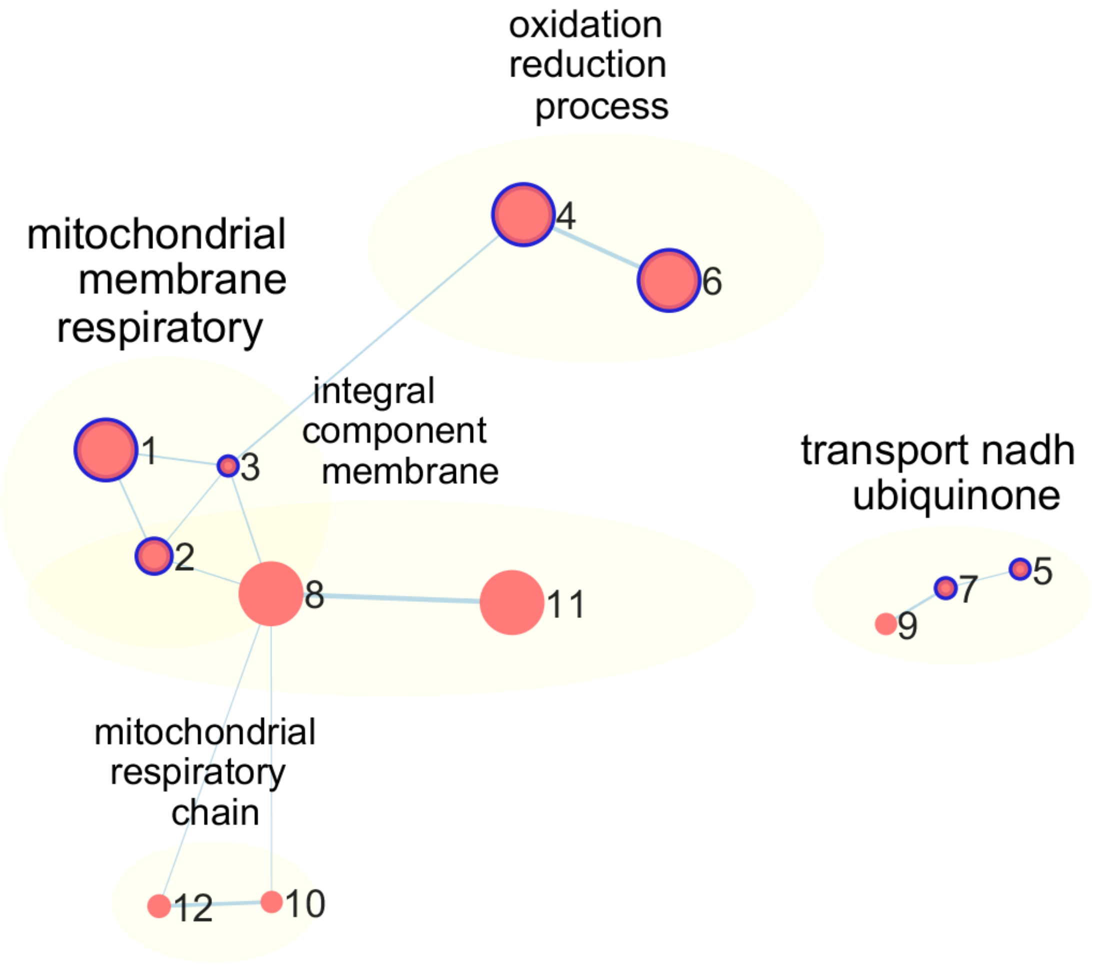


**Supplementary Figure 10 – Clusters of gene ontology (GO) categories enriched in 'Module 13'.** The applications 'EnrichmentMap'' (v3.2.0) and AutoAnnotate' (v1.3) in Cytoscape (v3.7.1) were used to generate this figure. GO terms are ordered by enrichment *P*-value with smaller numbers representing smaller *P*-values. GO terms were clustered by 'gene set description' ('EnrichmentMap'). Overrepresented GO terms are circled with a blue border (Bonferroni *P* < 3.34E-05, see **Supplementary Table 6**). The width of the edges is proportional to the number of genes in common between GO terms.

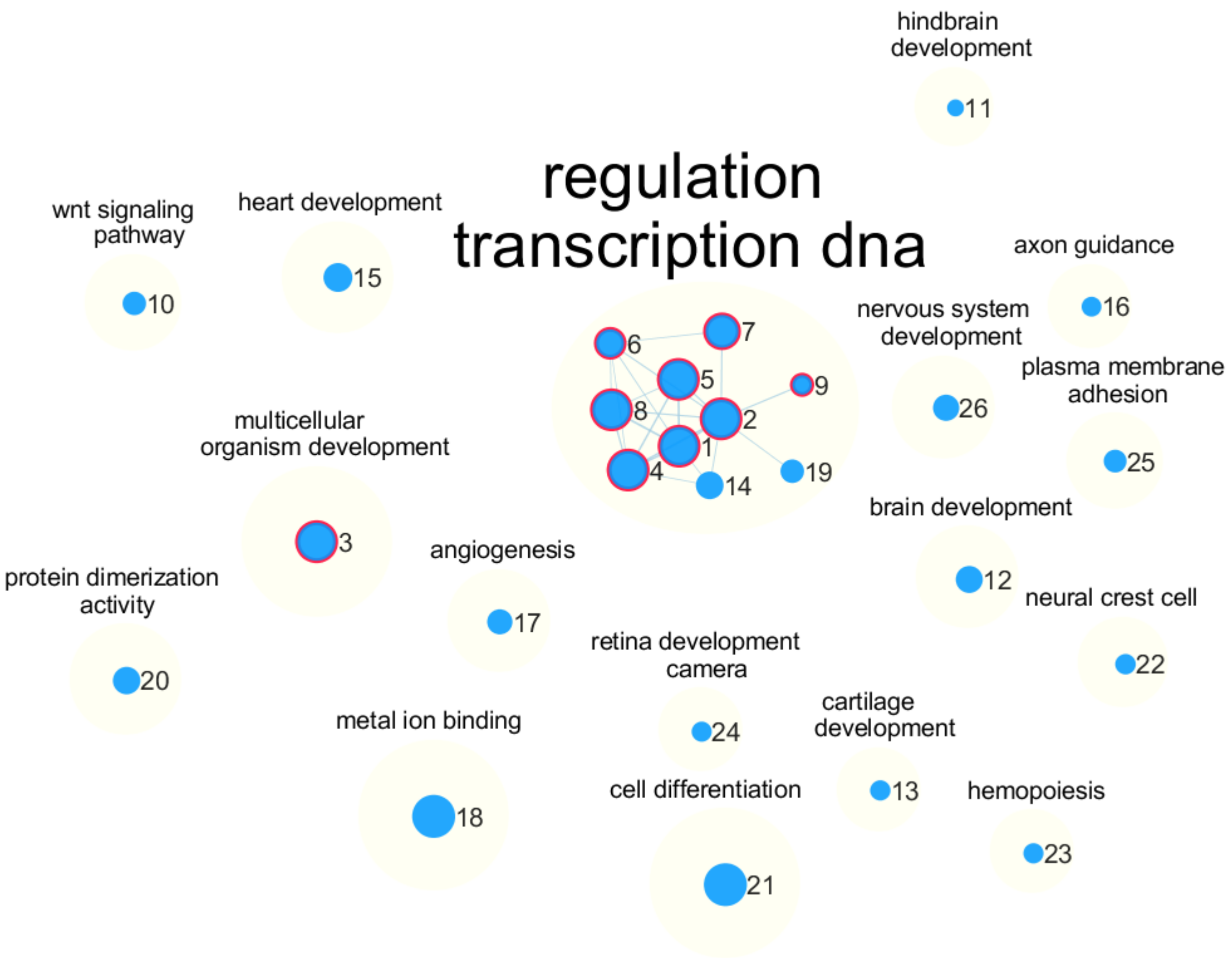


**Supplementary Figure 11 – Clusters of gene ontology (GO) categories enriched in 'Module 16'.** The applications 'EnrichmentMap'' (v3.2.0) and AutoAnnotate' (v1.3) in Cytoscape (v3.7.1) were used to generate this figure. GO terms are ordered by enrichment *P*-value with smaller numbers representing smaller *P*-values. GO terms were clustered by 'gene set description' ('EnrichmentMap'). Overrepresented GO terms are circled with a blue border (Bonferroni *P* < 3.34E-05, see **Supplementary Table 6**). The width of the edges is proportional to the number of genes in common between GO terms.

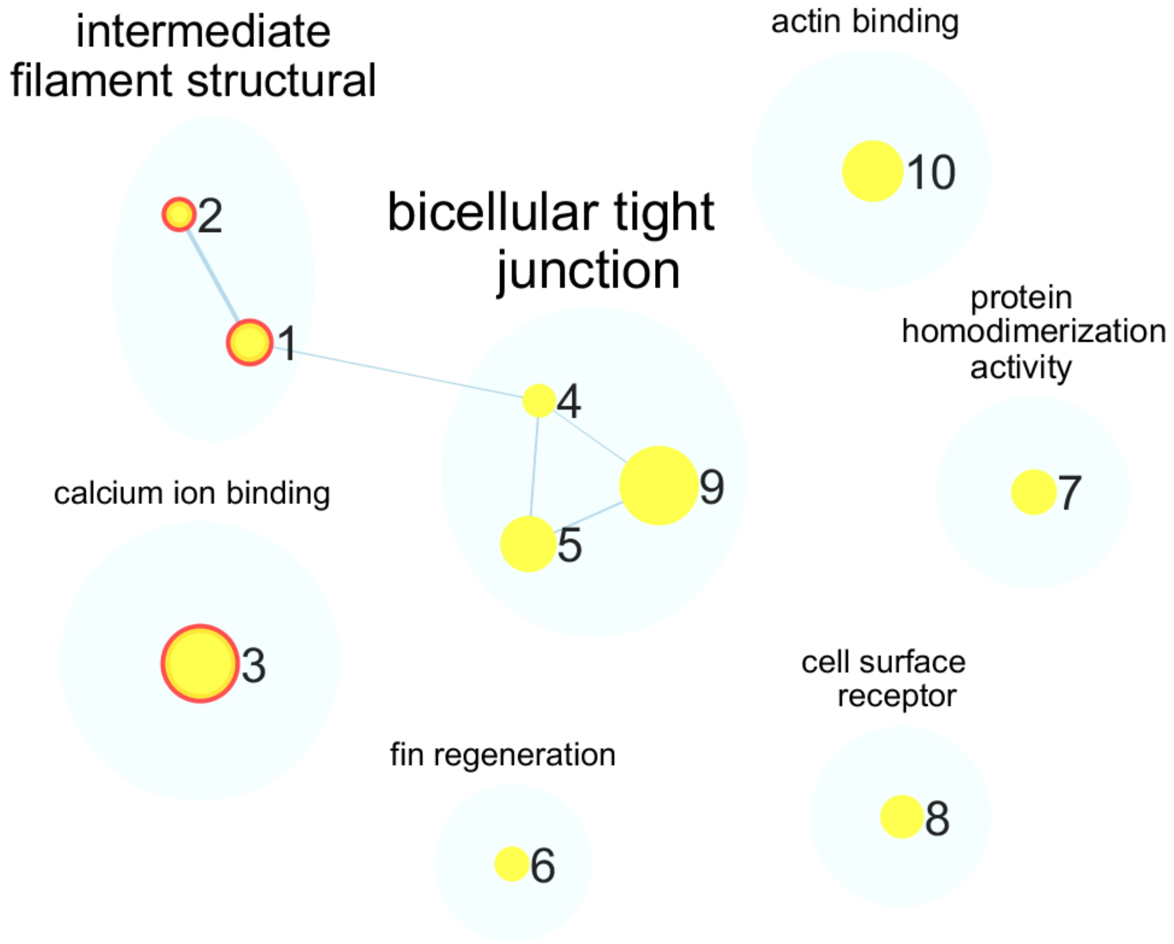


**Supplementary Figure 12 – Clusters of gene ontology (GO) categories enriched in 'Module 19'.** The applications 'EnrichmentMap'' (v3.2.0) and AutoAnnotate' (v1.3) in Cytoscape (v3.7.1) were used to generate this figure. GO terms are ordered by enrichment *P*-value with smaller numbers representing smaller *P*-values. GO terms were clustered by 'gene set description' ('EnrichmentMap'). Overrepresented GO terms are circled with a red border (Bonferroni $P < 3.34E\text{-}05$, see **Supplementary Table 6**). The width of the edges is proportional to the number of genes in common between GO terms.

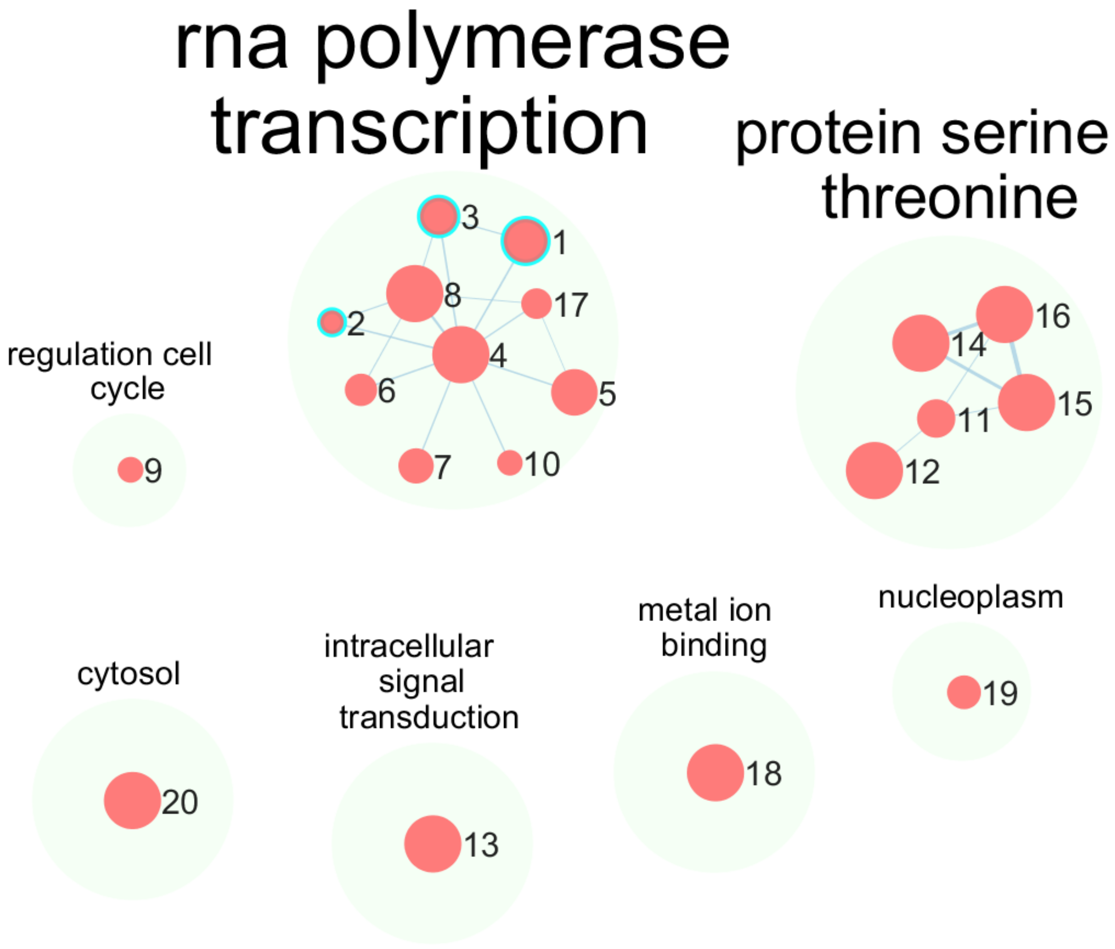


**Supplementary Figure 13 – Clusters of gene ontology (GO) categories enriched in 'Module 13'.** The applications 'EnrichmentMap'' (v3.2.0) and AutoAnnotate' (v1.3) in Cytoscape (v3.7.1) were used to generate this figure. GO terms are ordered by enrichment *P*-value with smaller numbers representing smaller *P*-values. GO terms were clustered by 'gene set description' ('EnrichmentMap'). Overrepresented GO terms are circled with a blue border (Bonferroni $P < 3.34E-05$, see **Supplementary Table 6**). The width of the edges is proportional to the number of genes in common between GO terms.

**Supplementary Table 1 – Phenotypic data for each of the samples used in this study.** Shown is the body weight (g), fork length (cm), liver weight (mg) and brain weight (mg), Fulton's condition factor (*k*), hepatosomatic index (HSI), brain-body ratio (BBR) and RNA integrity number (RIN) for each individual fish (see **Methods**).

| Fish ID | Exposure | Sex | Week | Tank | Body weight (g) | Length (cm) | Condition factor | Brain (mg) | Brain-body ratio | Liver (mg) | Hepatosomatic index | RIN |
|---|---|---|---|---|---|---|---|---|---|---|---|---|
| 1 | high | F | 2 | 5 | 0.36 | 3.30 | 0.99 | 4.81 | 1.35 | 8.64 | 2.42 | 9.4 |
| 2 | high | M | 1 | 5 | 0.42 | 3.40 | 1.06 | 5.87 | 1.4 | 1.79 | 0.43 | 9.2 |
| 3 | DMSO | M | 1 | 4 | 0.45 | 3.40 | 1.13 | 6.33 | 1.42 | 5.01 | 1.12 | 9.5 |
| 4 | low | F | 2 | 3 | 0.32 | 3.00 | 1.19 | 4.68 | 1.46 | 7.01 | 2.19 | 9.5 |
| 5 | high | F | 1 | 5 | 0.31 | 3.00 | 1.13 | 3.22 | 1.05 | 5.81 | 1.9 | 9.1 |
| 6 | DMSO | F | 1 | 6 | 0.54 | 3.50 | 1.26 | 5.34 | 0.99 | 11.9 | 2.21 | 9.3 |
| 7 | DMSO | F | 1 | 4 | 0.37 | 3.20 | 1.14 | 4.6 | 1.23 | 13.16 | 3.52 | 9.3 |
| 8 | water | M | 1 | 8 | 0.35 | 3.30 | 0.97 | 5.51 | 1.57 | 1.51 | 0.43 | 9.2 |
| 9 | low | F | 1 | 1 | 0.34 | 3.10 | 1.15 | 4.33 | 1.26 | 4.25 | 1.24 | 9.2 |
| 10 | DMSO | F | 2 | 4 | 0.34 | 3.20 | 1.02 | 4.23 | 1.26 | 9.13 | 2.73 | 9.3 |
| 11 | high | M | 1 | 7 | 0.36 | 3.40 | 0.92 | 4.64 | 1.28 | 2.35 | 0.65 | 9.3 |
| 12 | low | M | 1 | 1 | 0.39 | 3.50 | 0.91 | 5.95 | 1.53 | 1.7 | 0.44 | 9.9 |
| 13 | water | F | 2 | 8 | 0.32 | 3.10 | 1.09 | 5.19 | 1.6 | 6.97 | 2.15 | 9 |
| 14 | low | F | 1 | 3 | 0.36 | 3.10 | 1.21 | 4.88 | 1.36 | 3.24 | 0.9 | 9.4 |
| 15 | low | M | 1 | 1 | 0.5 | 3.20 | 1.52 | 5.52 | 1.11 | 1.15 | 0.23 | 9.3 |
| 16 | high | M | 1 | 5 | 0.34 | 3.30 | 0.94 | 5.61 | 1.65 | 1.25 | 0.37 | 9.4 |
| 17 | DMSO | M | 1 | 6 | 0.34 | 3.10 | 1.15 | 5.83 | 1.7 | 2.21 | 0.64 | 9.7 |
| 18 | water | M | 2 | 8 | 0.41 | 3.50 | 0.96 | 5.3 | 1.29 | 1.98 | 0.48 | 9.3 |
| 19 | water | F | 1 | 8 | 0.34 | 3.10 | 1.14 | 4.62 | 1.36 | 3.86 | 1.14 | 9.4 |
| 20 | high | M | 2 | 5 | 0.34 | 3.20 | 1.04 | 3.88 | 1.14 | 2.43 | 0.71 | 9.6 |
| 21 | DMSO | M | 2 | 4 | 0.52 | 3.60 | 1.11 | 6.55 | 1.26 | 3.3 | 0.64 | 8.7 |
| 22 | DMSO | F | 1 | 4 | 0.36 | 3.20 | 1.10 | 5.5 | 1.53 | 10.18 | 2.83 | 10 |
| 23 | high | M | 2 | 5 | 0.32 | 3.20 | 0.98 | 5.59 | 1.74 | 1.22 | 0.38 | 9.9 |
| 24 | low | M | 2 | 1 | 0.42 | 3.50 | 0.98 | 5.97 | 1.42 | 1.16 | 0.28 | 9.4 |
| 25 | DMSO | M | 2 | 6 | 0.38 | 3.40 | 0.97 | 5.9 | 1.55 | 1.71 | 0.45 | 9.1 |

| | | | | | | | | | | | | |
|---|---|---|---|---|---|---|---|---|---|---|---|---|
| 26 | water | M | 2 | 2 | 0.42 | 3.40 | 1.08 | NA | NA | 5.14 | 1.21 | 9.7 |
| 27 | water | M | 2 | 8 | 0.37 | 3.40 | 0.94 | 5.87 | 1.59 | 1.56 | 0.42 | 9.8 |
| 28 | low | M | 1 | 3 | 0.36 | 3.20 | 1.08 | 5.57 | 1.57 | 1.72 | 0.48 | 9.6 |
| 29 | water | F | 2 | 8 | 0.41 | 3.30 | 1.14 | 5.62 | 1.38 | 12.6 | 3.09 | 9.6 |
| 30 | water | M | 1 | 8 | 0.39 | 3.30 | 1.09 | 5.46 | 1.39 | 2.88 | 0.73 | 9.6 |
| 31 | DMSO | M | 2 | 4 | 0.37 | 3.30 | 1.02 | 6.35 | 1.73 | 1.3 | 0.35 | 9.9 |
| 32 | high | F | 1 | 7 | 0.41 | 3.40 | 1.04 | 5.25 | 1.28 | 10.09 | 2.46 | 9.4 |
| 33 | DMSO | F | 2 | 6 | 0.42 | 3.50 | 0.99 | 5.75 | 1.36 | 8.89 | 2.1 | - |
| 34 | water | M | 2 | 2 | 0.33 | 3.20 | 1.01 | 6.1 | 1.84 | 2.02 | 0.61 | 9.8 |
| 35 | high | F | 2 | 7 | 0.35 | 3.00 | 1.28 | 4.87 | 1.41 | 11.54 | 3.34 | 9.2 |
| 36 | high | M | 2 | 7 | 0.34 | 3.40 | 0.85 | 3.61 | 1.08 | 2.26 | 0.67 | 9.3 |
| 37 | water | F | 2 | 2 | 0.44 | 3.40 | 1.12 | 4.16 | 0.95 | 12.91 | 2.93 | 9.4 |
| 38 | high | F | 2 | 5 | 0.36 | 3.20 | 1.10 | 4.66 | 1.29 | 9.03 | 2.51 | 9 |
| 39 | water | F | 1 | 2 | 0.37 | 3.10 | 1.24 | 4.64 | 1.25 | 10.68 | 2.89 | 9.1 |
| 40 | water | M | 1 | 2 | 0.37 | 3.30 | 1.03 | 6.54 | 1.76 | 3.45 | 0.93 | 9.2 |
| 41 | water | F | 1 | 8 | 0.47 | 3.40 | 1.19 | 4.54 | 0.97 | 11.56 | 2.48 | 9.1 |
| 42 | low | F | 2 | 3 | - | 3.00 | - | 2.64 | - | 10.8 | - | 9.4 |
| 43 | water | F | 1 | 2 | 0.35 | 3.10 | 1.16 | 5.46 | 1.58 | 7.84 | 2.27 | 9.3 |
| 44 | DMSO | F | 2 | 6 | 0.38 | 3.35 | 1.01 | 5.62 | 1.48 | 9.01 | 2.37 | 9.8 |
| 45 | low | F | 1 | 1 | 0.35 | 3.20 | 1.07 | 4.1 | 1.17 | 10.72 | 3.05 | 9.8 |
| 46 | low | M | 2 | 3 | 0.45 | 3.50 | 1.05 | 6.37 | 1.42 | 3.1 | 0.69 | 9.3 |
| 47 | DMSO | F | 2 | 4 | 0.46 | 3.40 | 1.16 | 5.48 | 1.2 | 10.71 | 2.34 | 9.2 |
| 48 | DMSO | M | 1 | 6 | 0.45 | 3.70 | 0.89 | 5.64 | 1.25 | 4.42 | 0.98 | 9.3 |
| 49 | low | M | 2 | 3 | 0.41 | 3.50 | 0.95 | 5.24 | 1.29 | 1.98 | 0.49 | 9.4 |
| 50 | low | F | 2 | 1 | 0.32 | 3.10 | 1.07 | 4.49 | 1.41 | 9.08 | 2.85 | 9.3 |
| 51 | high | F | 2 | 7 | 0.32 | 3.10 | 1.07 | 4.68 | 1.47 | 8.89 | 2.79 | 9.3 |
| 52 | high | M | 1 | 7 | 0.43 | 3.40 | 1.09 | 1.77 | 0.41 | 3.6 | 0.84 | 9.4 |
| 53 | water | M | 1 | 2 | 0.39 | 3.40 | 1.00 | 5.56 | 1.41 | 1.9 | 0.48 | 8.3 |
| 54 | low | M | 1 | 3 | 0.26 | 3.00 | 0.98 | 5.1 | 1.93 | 2.62 | 0.99 | 9.5 |
| 55 | high | F | 1 | 7 | 0.53 | 3.60 | 1.13 | 5.87 | 1.12 | 11.95 | 2.27 | 9.4 |
| 56 | low | F | 2 | 1 | 0.43 | 3.30 | 1.19 | 6.54 | 1.53 | 22.83 | 5.35 | 9.3 |
| 57 | high | M | 2 | 7 | 0.37 | 3.50 | 0.87 | 5.69 | 1.53 | 1.29 | 0.35 | 9.5 |

| | | | | | | | | | | | | |
|---|---|---|---|---|---|---|---|---|---|---|---|---|
| 58 | water | F | 2 | 2 | 0.38 | 3.40 | 0.96 | 4.15 | 1.09 | 6.78 | 1.79 | 9.2 |
| 59 | DMSO | M | 2 | 6 | 0.4 | 3.50 | 0.94 | 6.4 | 1.58 | 1.95 | 0.48 | 9.4 |
| 60 | high | F | 1 | 5 | 0.43 | 3.40 | 1.08 | 4.64 | 1.09 | 10.5 | 2.46 | 9.4 |
| 61 | low | M | 2 | 1 | 0.32 | 3.30 | 0.89 | 5.58 | 1.74 | 0.54 | 0.17 | 8.7 |
| 62 | low | F | 1 | 3 | 0.42 | 3.30 | 1.15 | 4.42 | 1.07 | 8.85 | 2.13 | 9.3 |
| 63 | DMSO | F | 1 | 6 | 0.35 | 3.10 | 1.16 | 5.51 | 1.6 | 8.83 | 2.56 | 9.4 |
| 64 | DMSO | M | 1 | 4 | 0.33 | 3.30 | 0.93 | 5.78 | 1.73 | 2.51 | 0.75 | 9.6 |

**Supplementary Table 2 – Exposure to clozapine is not associated with changes in any of the measured physiological characteristics.** The first two columns show t-test *P*-values comparing the water and DMSO samples for body weight (g), fork length (cm), Fulton's condition factor (*k*), hepatosomatic index (HSI) and brain-body ratio (BBR) (see **Methods**). The two last columns show the ANOVAs *P*-values comparing the DMSO, 'low' (20µg/L) and 'high' (70µg/L) exposure groups for body weight, fork length, *k*, HSI and BBR. These tests were done separately for males and females since the two sexes can present large differences in these measurements.

| | **t-test water vs DMSO *P*-value** | | **ANOVA DMSO, low and high *P*-value** | |
|---|---|---|---|---|
| | **Females** | **Males** | **Females** | **Males** |
| **Body weight** | 0.58 | 0.34 | 0.48 | 0.44 |
| **Fork length** | 0.35 | 0.42 | 0.15 | 0.63 |
| **k** | 0.48 | 0.85 | 0.52 | 0.55 |
| **HSI** | 0.43 | 0.92 | 0.99 | 0.21 |
| **BBR** | 0.64 | 0.82 | 0.60 | 0.25 |

**Supplementary Table 3 – Exposure to clozapine is associated with changes in tank position.** Shown are the estimates, standard deviations (SD) and *P*-values of an analysis comparing the time spent in the top, middle or bottom of the tank during spawning, feeding and 'general time' (see **Methods**). **A)** shows the results of the linear regression comparing the water and DMSO samples (see **Methods**) and **B)** shows the ANOVA results comparing the DMSO, 'low' (20µg/L) and 'high' (70µg/L) exposure groups (see **Methods**). Highlighted are all *P*-values < 2.78E-03 (Bonferroni corrected *P*-value).

| | | | **Spawning** | **Feeding** | **'General time'** |
|---|---|---|---|---|---|
| **A) DMSO vs water** | **Top** | **Estimate** | 1.79 | 1.50 | 1.60 |
| | | **SD** | 0.91 | 1.34 | 1.30 |
| | | ***P*-value** | 0.05 | 0.27 | 0.22 |
| | **Middle** | **Estimate** | 0.69 | 1.31 | 0.65 |
| | | **SD** | 1.05 | 0.86 | 1.38 |
| | | ***P*-value** | 0.52 | 0.13 | 0.64 |
| | **Bottom** | **Estimate** | -2.48 | -2.81 | -2.25 |
| | | **SD** | 1.06 | 1.08 | 1.18 |
| | | ***P*-value** | 0.02 | 0.01 | 0.02 |
| **B) DMSO vs clozapine** | **Top** | **Estimate DMSO vs low** | 4.25 | 0.78 | 3.00 |
| | | **SD DMSO vs low** | 1.20 | 1.56 | 1.61 |
| | | **Estimate DMSO vs high** | 6.67 | 5.19 | 13.44 |
| | | **SD DMSO vs high** | 1.20 | 1.56 | 1.61 |
| | | ***P*-value** | 6.144E-07 | 2.41E-03 | 4.34E-14 |
| | **Middle** | **Estimate DMSO vs low** | 2.88 | 2.88 | 0.15 |
| | | **SD DMSO vs low** | 1.11 | 1.23 | 1.47 |
| | | **Estimate DMSO vs high** | -2.35 | -0.25 | -6.15 |
| | | **SD DMSO vs high** | 1.11 | 1.23 | 1.47 |
| | | ***P*-value** | 0.00003282 | 2.22E-02 | 1.55E-05 |
| | **Bottom** | **Estimate DMSO vs low** | -7.13 | -3.66 | -3.15 |
| | | **SD DMSO vs low** | 1.07 | 1.44 | 1.11 |

| | | | | | |
|---|---|---|---|---|---|
| | | **Estimate DMSO vs high** | -4.31 | -4.94 | -7.29 |
| | | **SD DMSO vs high** | 1.07 | 1.44 | 1.11 |
| | | ***P*-value** | 3.009E-09 | 2.69E-03 | 6.74E-09 |

**Supplementary Table 4 – RNA-seq quality control metrics for each sample.** Shown are the number of raw paired reads, number of paired reads after trimming (see **Methods**), percentage of trimmed paired reads in relation to the raw reads, number of mapped paired reads (see **Methods**), number of unique mapped reads (see **Methods**), percentage of unique mapped reads in relation to the raw reads and percentage of unique mapped reads in relation to the trimmed reads.

| Fish ID | Exposure | Raw paired reads | Trimmed paired reads | % Trimmed paired reads | Mapped paired reads | Unique mapped reads | % unique mapped reads (in relation to raw) | % unique mapped reads (in relation to trimmed) |
|---|---|---|---|---|---|---|---|---|
| 1 | high | 25,857,546 | 24,662,322 | 95.38 | 37,008,984 | 17,527,942 | 67.79 | 71.07 |
| 2 | high | 29,277,732 | 27,533,538 | 94.04 | 42,247,030 | 18,895,135 | 64.54 | 68.63 |
| 3 | DMSO | 34,694,030 | 32,859,138 | 94.71 | 49,884,656 | 23,037,310 | 66.40 | 70.11 |
| 4 | low | 21,518,362 | 20,173,874 | 93.75 | 30,558,882 | 14,366,569 | 66.76 | 71.21 |
| 5 | high | 23,459,864 | 22,511,080 | 95.96 | 32,875,582 | 16,301,997 | 69.49 | 72.42 |
| 6 | DMSO | 31,644,800 | 30,453,254 | 96.23 | 46,492,494 | 21,096,057 | 66.67 | 69.27 |
| 7 | DMSO | 43,804,656 | 41,519,558 | 94.78 | 61,731,348 | 29,616,887 | 67.61 | 71.33 |
| 8 | water | 43,474,072 | 41,061,480 | 94.45 | 61,755,966 | 29,187,557 | 67.14 | 71.08 |
| 9 | low | 43,709,854 | 41,160,864 | 94.17 | 60,579,514 | 30,008,517 | 68.65 | 72.91 |
| 10 | DMSO | 35,973,536 | 33,896,850 | 94.23 | 49,846,278 | 24,989,167 | 69.47 | 73.72 |
| 11 | high | 28,230,320 | 26,304,930 | 93.18 | 37,954,754 | 19,560,894 | 69.29 | 74.36 |
| 12 | low | 29,433,220 | 28,025,862 | 95.22 | 41,707,564 | 20,030,657 | 68.05 | 71.47 |
| 13 | water | 39,203,248 | 36,341,816 | 92.70 | 52,733,382 | 27,635,028 | 70.49 | 76.04 |
| 14 | low | 63,776,598 | 60,025,432 | 94.12 | 85,328,040 | 45,804,318 | 71.82 | 76.31 |
| 15 | low | 65,079,992 | 61,901,314 | 95.12 | 91,238,284 | 45,178,397 | 69.42 | 72.98 |
| 16 | high | 35,014,620 | 33,297,084 | 95.09 | 50,451,594 | 23,840,798 | 68.09 | 71.60 |
| 17 | DMSO | 39,428,424 | 37,792,988 | 95.85 | 55,874,152 | 27,389,566 | 69.47 | 72.47 |
| 18 | water | 52,533,504 | 49,327,384 | 93.90 | 72,432,912 | 36,462,701 | 69.41 | 73.92 |
| 19 | water | 58,565,430 | 54,933,806 | 93.80 | 80,230,594 | 40,944,996 | 69.91 | 74.54 |
| 20 | high | 38,981,036 | 36,706,200 | 94.16 | 54,189,878 | 26,745,441 | 68.61 | 72.86 |
| 21 | DMSO | 38,702,598 | 37,192,566 | 96.10 | 56,776,242 | 26,016,757 | 67.22 | 69.95 |
| 22 | DMSO | 37,677,664 | 35,859,700 | 95.17 | 52,838,662 | 26,273,738 | 69.73 | 73.27 |
| 23 | high | 26,744,744 | 25,219,084 | 94.30 | 36,257,074 | 18,961,647 | 70.90 | 75.19 |
| 24 | low | 37,499,686 | 35,636,388 | 95.03 | 52,651,544 | 25,816,288 | 68.84 | 72.44 |
| 25 | DMSO | 32,257,462 | 31,104,776 | 96.43 | 46,975,346 | 22,100,413 | 68.51 | 71.05 |

| | | | | | | | | |
|---|---|---|---|---|---|---|---|---|
| 26 | water | 43,007,104 | 40,936,332 | 95.19 | 60,910,762 | 29,604,253 | 68.84 | 72.32 |
| 27 | water | 34,217,002 | 31,700,266 | 92.64 | 46,029,848 | 23,955,635 | 70.01 | 75.57 |
| 28 | low | 43,805,868 | 41,437,066 | 94.59 | 61,875,764 | 29,973,172 | 68.42 | 72.33 |
| 29 | water | 32,600,568 | 30,987,834 | 95.05 | 45,659,546 | 22,569,765 | 69.23 | 72.83 |
| 30 | water | 38,364,790 | 36,903,484 | 96.19 | 54,680,470 | 26,596,424 | 69.33 | 72.07 |
| 31 | DMSO | 30,440,606 | 29,446,940 | 96.74 | 45,207,270 | 20,233,804 | 66.47 | 68.71 |
| 32 | high | 36,037,706 | 34,342,716 | 95.30 | 51,598,372 | 24,654,776 | 68.41 | 71.79 |
| 33 | DMSO | 45,754,756 | 43,770,602 | 95.66 | 66,152,026 | 31,080,604 | 67.93 | 71.01 |
| 34 | water | 32,877,826 | 31,427,486 | 95.59 | 46,725,488 | 22,736,280 | 69.15 | 72.35 |
| 35 | high | 31,701,704 | 30,470,952 | 96.12 | 45,387,846 | 21,873,404 | 69.00 | 71.78 |
| 36 | high | 28,296,630 | 27,185,114 | 96.07 | 40,541,694 | 19,366,933 | 68.44 | 71.24 |
| 37 | water | 22,987,042 | 21,881,980 | 95.19 | 31,462,580 | 16,616,358 | 72.29 | 75.94 |
| 38 | high | 36,318,806 | 34,976,864 | 96.31 | 53,114,016 | 24,678,636 | 67.95 | 70.56 |
| 39 | water | 32,874,574 | 31,376,030 | 95.44 | 46,693,730 | 22,796,292 | 69.34 | 72.66 |
| 40 | water | 21,305,308 | 20,303,120 | 95.30 | 30,468,102 | 14,517,787 | 68.14 | 71.51 |
| 41 | water | 28,545,900 | 27,438,164 | 96.12 | 41,665,210 | 19,260,109 | 67.47 | 70.19 |
| 42 | low | 29,125,062 | 27,867,122 | 95.68 | 42,000,128 | 19,757,655 | 67.84 | 70.90 |
| 43 | water | 25,006,472 | 23,948,084 | 95.77 | 35,329,690 | 17,311,313 | 69.23 | 72.29 |
| 44 | DMSO | 26,048,126 | 24,313,206 | 93.34 | 35,482,884 | 17,920,900 | 68.80 | 73.71 |
| 45 | low | 38,438,124 | 36,608,946 | 95.24 | 54,487,298 | 26,115,517 | 67.94 | 71.34 |
| 46 | low | 39,976,674 | 38,287,674 | 95.78 | 58,212,496 | 26,829,398 | 67.11 | 70.07 |
| 47 | DMSO | 35,373,872 | 33,772,764 | 95.47 | 51,438,670 | 23,840,770 | 67.40 | 70.59 |
| 48 | DMSO | 34,040,346 | 32,435,878 | 95.29 | 49,241,686 | 22,719,156 | 66.74 | 70.04 |
| 49 | low | 38,126,574 | 35,946,182 | 94.28 | 52,890,314 | 26,275,643 | 68.92 | 73.10 |
| 50 | low | 32,346,608 | 30,768,784 | 95.12 | 46,715,012 | 21,811,313 | 67.43 | 70.89 |
| 51 | high | 31,933,598 | 30,460,620 | 95.39 | 46,314,106 | 21,594,175 | 67.62 | 70.89 |
| 52 | high | 35,180,150 | 33,455,112 | 95.10 | 50,202,962 | 23,812,598 | 67.69 | 71.18 |
| 53 | water | 29,334,058 | 28,191,686 | 96.11 | 42,444,174 | 19,943,165 | 67.99 | 70.74 |
| 54 | low | 38,371,786 | 36,799,228 | 95.90 | 54,848,428 | 26,156,217 | 68.17 | 71.08 |
| 55 | high | 36,221,064 | 34,091,048 | 94.12 | 50,027,088 | 25,133,205 | 69.39 | 73.72 |
| 56 | low | 20,950,978 | 19,997,362 | 95.45 | 30,237,116 | 14,096,291 | 67.28 | 70.49 |
| 57 | high | 39,361,098 | 37,476,884 | 95.21 | 56,630,282 | 26,499,770 | 67.32 | 70.71 |
| 58 | water | 29,706,856 | 28,032,714 | 94.36 | 41,729,320 | 20,370,469 | 68.57 | 72.67 |
| 59 | DMSO | 40,946,892 | 39,231,642 | 95.81 | 59,810,494 | 27,546,919 | 67.27 | 70.22 |

| | | | | | | | | |
|---|---|---|---|---|---|---|---|---|
| 60 | high | 27,910,182 | 26,677,934 | 95.58 | 40,714,560 | 18,502,238 | 66.29 | 69.35 |
| 61 | low | 26,755,496 | 25,640,246 | 95.83 | 39,204,916 | 17,755,336 | 66.36 | 69.25 |
| 62 | low | 25,983,956 | 24,622,534 | 94.76 | 38,058,160 | 17,048,741 | 65.61 | 69.24 |
| 63 | DMSO | 33,235,704 | 31,198,978 | 93.87 | 46,053,836 | 23,095,418 | 69.49 | 74.03 |
| 64 | DMSO | 18,817,392 | 17,791,292 | 94.55 | 27,052,300 | 12,422,219 | 66.01 | 69.82 |

**Supplementary Table 5 – Gene ontology (GO) categories enriched in each of the gene co-expression networks associated with clozapine exposure.** 'Expressed genes in GO' shows how many genes expressed in our dataset are in that GO term and 'Module gene in GO' shows how many module genes are in that GO term.

| Module | GO category | Sub-ontology | Description | Over representation *P* | Expressed genes in GO | Module gene in GO |
|---|---|---|---|---|---|---|
| 1 | GO:0004930 | MF | G protein-coupled receptor activity | 1.56E-06 | 47 | 21 |
| | GO:0007165 | BP | signal transduction | 8.58E-06 | 114 | 42 |
| | GO:0007186 | BP | G protein-coupled receptor signaling pathway | 1.26E-05 | 60 | 23 |
| 4 | GO:0015986 | BP | ATP synthesis coupled proton transport | 9.5944E-14 | 15 | 13 |
| | GO:0046933 | MF | proton-transporting ATP synthase activity, rotational mechanism | 1.26096E-13 | 13 | 12 |
| | GO:0005739 | CC | mitochondrion | 6.21472E-11 | 77 | 23 |
| | GO:0005743 | CC | mitochondrial inner membrane | 4.96861E-10 | 32 | 15 |
| | GO:0004129 | MF | cytochrome-c oxidase activity | 1.79586E-07 | 15 | 9 |
| | GO:0005747 | CC | mitochondrial respiratory chain complex I | 6.59292E-07 | 17 | 9 |
| | GO:0015078 | MF | proton transmembrane transporter activity | 2.78583E-06 | 11 | 7 |
| | GO:0005751 | CC | mitochondrial respiratory chain complex IV | 2.58042E-05 | 10 | 6 |
| 5 | GO:0002181 | BP | cytoplasmic translation | < 2.93E-98 | 15 | 15 |
| | GO:0022625 | CC | cytosolic large ribosomal subunit | < 2.93E-98 | 39 | 39 |
| | GO:0022627 | CC | cytosolic small ribosomal subunit | < 2.93E-98 | 28 | 28 |
| | GO:0005840 | CC | ribosome | 2.93E-98 | 81 | 80 |
| | GO:0003735 | MF | structural constituent of ribosome | 5.09E-92 | 79 | 77 |
| | GO:0006412 | BP | translation | 2.09E-86 | 93 | 79 |
| | GO:0005622 | CC | intracellular | 2.32E-55 | 81 | 60 |
| | GO:0043009 | BP | chordate embryonic development | 1.31E-15 | 34 | 19 |
| | GO:0051726 | BP | regulation of cell cycle | 2.47E-08 | 17 | 11 |

| | | | | | | |
|---|---|---|---|---|---|---|
| | GO:0030218 | BP | erythrocyte differentiation | 2.90E-08 | 12 | 9 |
| | GO:0003723 | MF | RNA binding | 4.00E-08 | 63 | 19 |
| | GO:0006414 | BP | translational elongation | 4.86E-06 | 10 | 6 |
| **7** | GO:0016020 | CC | membrane | 2.06E-05 | 580 | 36 |
| **8** | GO:0003676 | MF | nucleic acid binding | 1.32E-18 | 104 | 27 |
| | GO:0003723 | MF | RNA binding | 1.49E-17 | 63 | 22 |
| | GO:0000381 | BP | regulation of alternative mRNA splicing, via spliceosome | 2.79E-12 | 12 | 9 |
| | GO:0003729 | MF | mRNA binding | 3.83E-10 | 20 | 10 |
| | GO:0008380 | BP | RNA splicing | 5.65E-08 | 13 | 7 |
| | GO:0006397 | BP | mRNA processing | 1.97E-07 | 15 | 7 |
| **11** | GO:0009416 | BP | response to light stimulus | 8.22E-09 | 19 | 8 |
| | GO:0000976 | MF | transcription regulatory region sequence-specific DNA binding | 1.49E-08 | 22 | 8 |
| | GO:0008134 | MF | transcription factor binding | 4.63E-08 | 16 | 7 |
| | GO:0032922 | BP | circadian regulation of gene expression | 2.26E-07 | 12 | 6 |
| | GO:0001078 | MF | proximal promoter DNA-binding transcription repressor activity, RNA polymerase II-specific | 1.80E-06 | 16 | 6 |
| | GO:0003700 | MF | DNA-binding transcription factor activity | 2.00E-06 | 110 | 13 |
| | GO:0005634 | CC | nucleus | 1.24E-05 | 389 | 23 |
| | GO:0000122 | BP | negative regulation of transcription by RNA polymerase II | 1.41E-05 | 24 | 6 |
| | GO:0006351 | BP | transcription, DNA-templated | 1.43E-05 | 36 | 7 |
| **13** | GO:0005739 | CC | mitochondrion | < 2.93E-98 | 77 | 19 |
| | GO:0005743 | CC | mitochondrial inner membrane | < 2.93E-98 | 32 | 13 |
| | GO:0070469 | CC | respiratory chain | < 2.93E-98 | 19 | 14 |
| | GO:0055114 | BP | oxidation-reduction process | 3.54E-10 | 77 | 15 |
| | GO:0008137 | MF | NADH dehydrogenase (ubiquinone) activity | 5.43E-08 | 13 | 8 |
| | GO:0016491 | MF | oxidoreductase activity | 6.70E-07 | 62 | 11 |

| | | | | | | |
|---|---|---|---|---|---|---|
| | GO:0005747 | CC | mitochondrial respiratory chain complex I | 1.48E-06 | 17 | 7 |
| **16** | GO:0006355 | BP | regulation of transcription, DNA-templated | < 2.93E-98 | 185 | 19 |
| | GO:0005634 | CC | nucleus | 2.65E-09 | 389 | 23 |
| | GO:0007275 | BP | multicellular organism development | 1.79E-08 | 93 | 12 |
| | GO:0003677 | MF | DNA binding | 2.37E-08 | 194 | 16 |
| | GO:0043565 | MF | sequence-specific DNA binding | 3.43E-08 | 124 | 13 |
| | GO:0000981 | MF | DNA-binding transcription factor activity, RNA polymerase II-specific | 1.17E-07 | 54 | 9 |
| | GO:0006357 | BP | regulation of transcription by RNA polymerase II | 2.61E-07 | 58 | 9 |
| | GO:0003700 | MF | DNA-binding transcription factor activity | 8.56E-06 | 110 | 10 |
| | GO:0000977 | MF | RNA polymerase II regulatory region sequence-specific DNA binding | 2.50E-05 | 37 | 6 |
| **19** | GO:0005198 | MF | structural molecule activity | < 2.93E-98 | 31 | 11 |
| | GO:0005882 | CC | intermediate filament | 3.60E-07 | 15 | 6 |
| | GO:0005509 | MF | calcium ion binding | 4.67E-06 | 75 | 7 |
| **25** | GO:0006357 | BP | regulation of transcription by RNA polymerase II | 6.03E-07 | 58 | 9 |
| | GO:0000978 | MF | RNA polymerase II proximal promoter sequence-specific DNA binding | 2.61E-06 | 24 | 6 |
| | GO:0000981 | MF | DNA-binding transcription factor activity, RNA polymerase II-specific | 8.47E-06 | 54 | 8 |
